# Superior Multimodal Luminescence in a Stable Single‐Host Nanomaterial with Large‐Scale Synthesis for High‐Level Anti‐Counterfeiting and Encryption

**DOI:** 10.1002/advs.202415473

**Published:** 2025-01-13

**Authors:** Bingyin Kong, Gencai Pan, Mengke Wang, Hongye Tang, Zhipeng Lv, Shiyu Sun, Yuxin Luo, Wenwu You, Wen Xu, Yanli Mao

**Affiliations:** ^1^ Key Laboratory for High Efficiency Energy Conversion Science and Technology of Henan Province International Joint Research Laboratory of New Energy Materials and Devices of Henan Province School of Physics and Electronics Henan University Kaifeng 475004 P. R. China; ^2^ Key Laboratory of New Energy and Rare Earth Resource Utilization of State Ethnic Affairs Commission School of Physics and Materials Engineering Dalian Minzu University Dalian 116600 P. R. China

**Keywords:** color‐tuning persistent luminescence, energy migration, hydrochromism, lanthanide‐based metal halide, self‐trapped exciton

## Abstract

Multimode luminescent materials exhibit tunable photon emissions under different excitation or stimuli channels, endowing them high encoding capacity and confidentiality for anti‐counterfeiting and encryption. Achieving multimode luminescence into a stable single material presents a promising but remains a challenge. Here, the downshifting/upconversion emissions, color‐tuning persistent luminescence (PersL), temperature‐dependent multi‐color emissions, and hydrochromism are integrated into Er^3+^ ions doped Cs_2_NaYbCl_6_ nanocrystals (NCs) by leveraging shallow defect levels and directed energy migration. The resulting NCs display strong static and dynamic colorful luminescence in response to ultraviolet, 980‐nm laser, and X‐ray. Additionally, the NCs exhibit distinct luminescent colors as the temperature increases from 330 to 430 K. Surprisingly, it also demonstrates the ability of the reversible emission modal and color in response to water. Theoretical calculations and experimental characterizations reveal that self‐trapped exciton state (STEs), chlorine vacancy defects, and ladderlike 4f energy levels of Er^3+^ ions contribute to multimodal luminescence. More importantly, it has extremely remarkable environmental stability, which can be stored in the air for more than 18 months, showing promising commercial prospects. This work not only gives new insights into lanthanide‐based metal halide NCs but also provides a new route for developing multimodal luminescent nanomaterials for anti‐counterfeiting and encryption.

## Graphical abstract

In this work, five‐mode luminescence (downshifting/upconversion dual‐color emissions, color‐tuning persistent luminescence (PersL), temperature‐dependent multi‐color emissions, and reversible hydrochromic emissions) are achieved in Er^3+^ ions doped Cs_2_NaYbCl_6_ NCs by leveraging self‐trapped exciton state (STEs), chlorine vacancies defects and directed energy migration. Finally, the advanced anti‐counterfeiting schemes and encryption strategies are designed and realized.

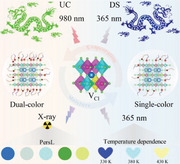

## 1 Introduction

Counterfeiting has become a global serious issue that can cause critical financial and health damage and pose significant risks to both individuals and society.^[^ ^1^ ^]^ In consideration of this, a series of anti‐counterfeiting technologies and strategies have been developed to enhance the security level of anti‐counterfeiting labels. Wherein, the luminescence anti‐counterfeiting is particularly concerned due to the fascinating optical properties of luminescent materials, such as visually identifiable emission colors, various emission lifetimes, and abundant emission modes in response to diverse excitation sources.^[^ ^2^ ^]^

In the past decades, commonly used luminescent materials for anti‐counterfeiting include carbon dots,^[^ ^3^ ^]^ nanocrystals (NCs),^[^ ^4^ ^]^ coordination polymers,^[^ ^5^ ^]^ metal halide perovskites,^[^ ^6^ ^]^ and lanthanide ions doped materials.^[^ ^7^ ^]^ However, traditional single materials generally have a single luminescent mode, which is easy to replicate and hinders their practical applications. To enhance the anti‐counterfeiting level, more smart luminescent materials have been developed by constructing composites, core–shell nanoparticles, or mixing materials, which can achieve multimode luminescence under different stimuli channels.^[^ ^8^ ^]^ For example, the composite composed of rare‐earth fluoride nanoparticles and quantum dots (QDs) can achieve dual‐mode luminescence of down shifting (DS) and upconversion (UC) under ultraviolet (UV)/near‐infrared (NIR) light excitation.^[^ ^9^ ^]^ The core/shell nanoparticles show tunable color changes under UV or/and NIR light excitation.^[^ ^10^ ^]^ A mixture of organic molecules and metal halides exhibits excitation‐dependent persistent luminescence, and the afterglow depends on multimodes including excitation, temperature, and time.^[^ ^11^ ^]^ Nevertheless, this type of material suffers from the inefficiencies and complexities associated with level mismatch and physicochemical incompatibility.^[^ ^12^ ^]^ So achieving multimode luminescence in a stable single material is a more promising strategy for advanced anti‐counterfeiting and encryption.

Trivalent lanthanide (Ln^3+^) ions doped inorganic phosphors have shown significant potential in achieving multi‐mode luminescence in a single materials, which benefits from the abundant 4f energy levels, wide emission wavelength range (UV–vis and NIR), and rich crystal structure of the host.^[^ ^13^ ^]^ However, this is not an easy task to integrate a series of luminescent modes into a single Ln^3+^ ions doped material, because the interactions between energy levels of Ln^3+^ ions and intrinsic or extrinsic defect levels of the host are complex. For example, the appropriate defect depths of the host are necessary to ensure the capture and release of charge carriers during or after the excitation, as well as transfer energy to Ln^3+^ ions in the lattice of the host. So it is still a challenging task to achieve the control of such defects, carrier capture or de‐capture, and response to external stimuli (water, heat, or mechanical forces) in a single Ln^3+^ ions doped material simultaneously.

Herein, we achieved five‐mode luminescence (DS/UC dual‐color emissions, color‐tuning persistent luminescence (PersL), temperature‐dependent color‐tuning emissions, and reversible hydrochromic emissions) in Er^3+^ ions doped Cs_2_NaYbCl_6_ NCs by leveraging self‐trapped exciton state (STEs), chlorine vacancies defects and directed energy migration. The intrinsic transition energy levels of Er^3+^ and Yb^3+^ ions provide the DS/UC dual‐mode emissions in response to UV or NIR light excitation. The temperature dependence luminescent is because the interaction between excitons and phonons strengthens as the temperature increases, resulting in a decrease in the excited state energy. The chlorine vacancy defects with shallow trap depths and cross relaxation between Er^3+^ ions create the color tunable afterglow after cessation of X‐ray irradiation. The reversible hydrochromic luminescence originates from the physicochemical nature of Er^3+^ ions doped Cs_2_NaYbCl_6_ NCs. The first‐principles calculations reveal the main intrinsic defects are chlorine vacancies, which play a vital role in dynamical luminescence processes. Finally, the advanced anti‐counterfeiting schemes and encryption strategies were designed and realized, demonstrating this nanomaterial has a high coding capacity and confidentiality. This new finding indicates that the target combination of Ln^3+^ ions and metal halide NCs will provide new opportunities for multi‐faceted applications.

## 2 Results and Discussion

**Figure 1:**
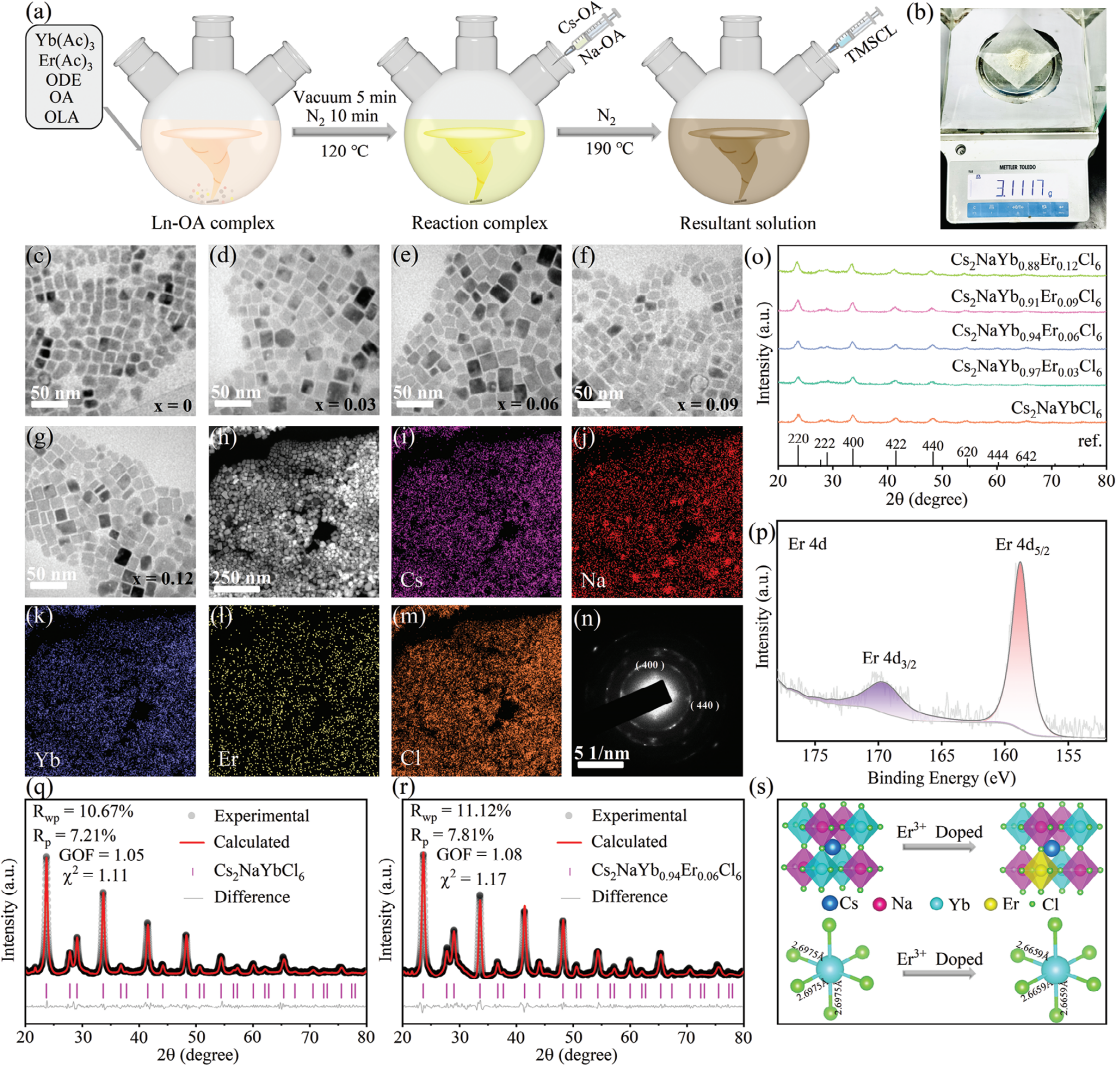
a) Schematic for the synthesis of Cs_2_NaYb_1‐x_Er_x_Cl_6_ (x = 0, 0.03, 0.06, 0.09, and 0.12) NCs. b) Weighing image of large‐scale synthesis of Cs_2_NaYb_0.94_Er_0.06_Cl_6_ NCs (≈3.1 g). c–g) TEM images of Cs_2_NaYb_1‐x_Er_x_Cl_6_ NCs. h–m) EDX mapping images of each element in Cs_2_NaYb_0.94_Er_0.06_Cl_6_ NCs. n) SAED pattern of Cs_2_NaYb_0.94_Er_0.06_Cl_6_ NCs. o) The XRD patterns of Cs_2_NaYb_1‐x_Er_x_Cl_6_ (x = 0, 0.03, 0.06, 0.09, and 0.12) NCs. p) High‐resolution XPS spectra of Er 4d. q,r) Rietveld refinement results of Cs_2_NaYbCl_6_ and Cs_2_NaYb_0.94_Er_0.06_Cl_6_ NCs. s) Typical crystal structure of metal halide octahedra and schematic diagram of the environmental changes for Yb‐Cl in Cs_2_NaYbCl_6_ and Cs_2_NaYb_0.94_Er_0.06_Cl_6_ NCs. (Crystal structure and Yb‐Cl obtained from Rietveld refinement result).

Undoped and Er^3+^ ions doped Cs_2_NaYbCl_6_ metal halide NCs are synthesized by a modified hot‐injection method (details in the Supporting Information). In a nutshell, Yb(Ac)_3_ and Er(Ac)_3_ with different dosages are foremost dissolved in octadecene (ODE) mixed with oleic acid (OA) and oleylamine (OLA) to form a precursor solution of Ln^3+^ ions. Moreover, NaAc and Cs_2_CO_3_ are respectively dissolved in ODE mixed with OA to produce Na‐oleate and Cs‐oleate solutions, which are respectively used as the injection stock solutions. Subsequently, Na‐oleate and Cs‐oleate solutions are first injected into the Ln^3+^ ions precursor at 120 °C. After 5 min, the solution is heated to 190 °C under a nitrogen atmosphere, and then chlorotrimethylsilane (TMS‐Cl) is swiftly injected again (**Figure** **1**a). After 1 min, the reaction is stopped with an ice bath. After purification, the NCs are obtained. This synthesis method underwent two injections to ensure sufficient dissolution of metal cations, achieving large‐scale synthesis (Figure 1b). The transmission electron microscopy (TEM) micrographs of Cs_2_NaYb_1‐x_Er_x_Cl_6_ (x = 0, 0.03, 0.06,0.09 and 0.12) NCs reveal the quadrilaterals with an average size of ≈14.7 nm (Figure 1c–g; Figure S1, Supporting Information). High‐resolution TEM (HR‐TEM) observations of Cs_2_NaYbCl_6_ and Cs_2_NaYb_0.94_Er_0.06_Cl_6_ NCs reveal that the two NCs possess legible crystal lattices with the lattice distances of 3.81 and 3.85 Å corresponding to the (220) plane (Figure S2, Supporting Information). The increase of the lattice constant confirms that larger Er^3+^ ions partially replace smaller Yb^3+^ ions. The energy‐dispersive X‐ray (EDX) analysis and mapping images further confirm the inclusion of Er^3+^ ions in Cs_2_NaYbCl_6_ NCs (Figure 1h–m; Figure S3, Supporting Information), and the inductively coupled plasma optical emission spectrometer (ICP‐OES) measurements show that the actual doping amount and feeding amount of Er^3+^ ions are almost the same (Table S1, Supporting Information). The selected area electron diffraction (SAED) pattern indicates that the NCs possess a cubic structure with corresponding (400) and (440) planes (Figure 1n). The X‐ray diffraction (XRD) patterns of Cs_2_NaYb_1‐x_Er_x_Cl_6_ (x = 0, 0.03, 0.06, 0.09, and 0.12) NCs further confirm the NCs are cubic phase, and the diffraction peaks shifted toward a smaller angle with the increase of Er^3+^ ions doping, indicating that the lattice expanded and further suggesting that smaller Yb^3+^ ions were partially replaced by larger Er^3+^ ions (Figure 1o). X‐ray photoelectron spectroscopy (XPS) comparative measurements reveal that Cs_2_NaYbCl_6_ and Cs_2_NaYb_0.94_Er_0.06_Cl_6_ NCs both comprise Cs, Na, Yb, and Cl elements (Figures S4a, S5a, Supporting Information). The high‐resolution XPS spectra reveal that the binding energies of Er^3+^ 4d_3/2_ and Er^3+^ 4d_5/2_ have emerged in Cs_2_NaYb_0.94_Er_0.06_Cl_6_ NCs (Figure 1p; Figures S4b–e, S5b–e, Supporting Information). The Rietveld refinement XRD patterns of Cs_2_NaYbCl_6_ and Cs_2_NaYb_0.94_Er_0.06_Cl_6_ NCs show that all diffraction peaks correspond well to the calculated Bragg positions, excluding impurity peaks (Figure 1q–r), while the bond length of Yb‐Cl of [YbCl_6_]^3‐^ decreases from 2.6975 to 2.6659 Å (Figure 1s; Table S2, Supporting Information), causing slight lattice distortion. These results above indicate the successful preparation of Cs_2_NaYb_1‐x_Er_x_Cl_6_ NCs.

**Figure 2:**
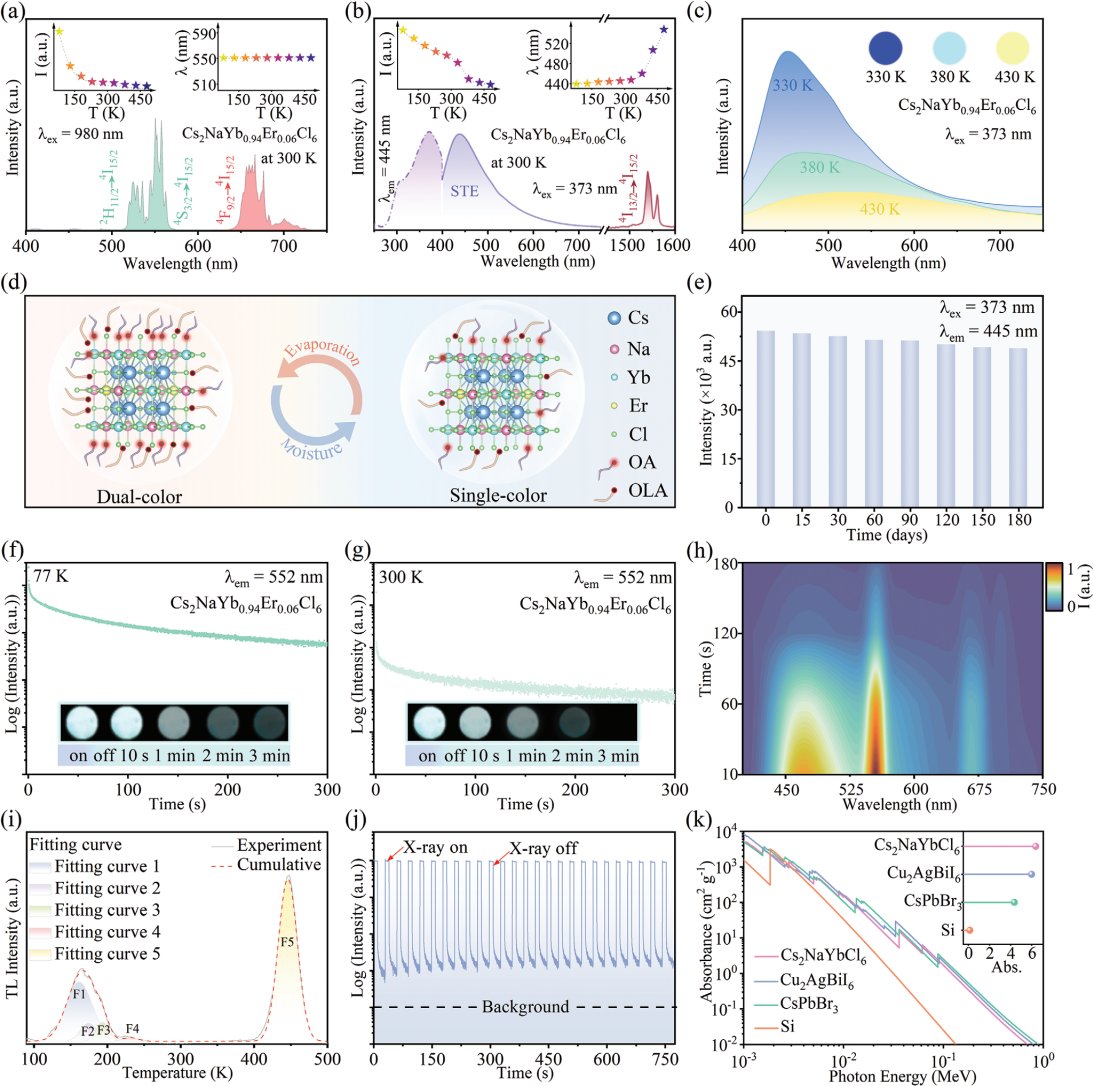
a) UC PL spectra of Cs_2_NaYb_0.94_Er_0.06_Cl_6_ NCs under 980 nm laser excitation at 300 K. The insets display the temperature‐dependent visible PL intensities and emission peak shift. b) Visible PL, NIR PL, and PLE spectra of Cs_2_NaYb_0.94_Er_0.06_Cl_6_ NCs at 300 K. The insets display the temperature‐dependent visible PL intensities and emission peak shift. c) Visible PL and optical photographs of Cs_2_NaYb_0.94_Er_0.06_Cl_6_ NCs under 373 nm excitation at 330, 380, and 430 K. d) A schematic diagram showing the reversible transformation of Cs_2_NaYb_0.94_Er_0.06_Cl_6_ NCs by humidifying‐drying cycle. e) Histogram showing maximum luminescence intensity of Cs_2_NaYb_0.94_Er_0.06_Cl_6_ NCs in aqueous solution at different time intervals. f,g) PersL photographs and decay curves of Cs_2_NaYb_0.94_Er_0.06_Cl_6_ NCs were captured after removing the X‐ray excitation source at 77 and 300 K. h) Contour plot of time‐dependent PersL spectra of Cs_2_NaYb_0.94_Er_0.06_Cl_6_ NCs after the cessation of X‐ray. i) TL spectra of Cs_2_NaYb_0.94_Er_0.06_Cl_6_ NCs. j) The photostability of Cs_2_NaYb_0.94_Er_0.06_Cl_6_ NCs under 60 kV X‐ray irradiation over 26 consecutive cycles (a 30 s interval between each cycle). k) Absorption spectra of Cs_2_NaYbCl_6_, Cu_2_AgBiI_6_, CsPbBr_3_, and Si as a function of X‐ray energy. The inset is compares of X‐ray absorption spectra at 60 kV for Cs_2_NaYbCl_6_, Cu_2_AgBiI_6_, CsPbBr_3_, and Si.

The multimode emission features have been studied successively. The UC luminescence spectra of Cs_2_NaYb_1‐x_Er_x_Cl_6_ (x = 0, 0.03, 0.06, 0.09, and 0.12) NCs under 980 nm laser excitation were recorded in Figure S6 (Supporting Information). The UC emission intensity reaches the maximum when the value of x is 0.06, the fluorescence decay lifetimes of Er^3+^ ions conform to this pattern of change (Figure S7a,b, Supporting Information). The optimal UC photoluminescence quantum yield (PLQY) of the samples is 3.52% (Figure S8, Supporting Information). The UC green emissions at ≈524 and 552 nm are derived from the ^2^H_11/2_→^4^I_15/2_ and ^4^S_3/2_→^4^I_15/2_ transitions of Er^3+^, while the UC red emission at ≈665 nm originates from ^4^F_9/2_→^4^I_15/2_ transition of Er^3+^ (**Figure** **2**a). As shown in the insets, the integral intensity of UC emission gradually decreases with the increase in temperature, while the emission wavelength has hardly changed (Figure S9, Supporting Information), which is consistent with the previously reported results of UC luminescence for Er^3+^ ions.^[^ ^14^ ^]^ Under 980 nm laser excitation, the near‐infrared (NIR) emission originating from ^4^I_13/2_→^4^I_15/2_ transition of Er^3+^ ions can be also observed (Figure S10, Supporting Information). The proposed mechanism diagram explains the electronic transition process of UC luminescence for the NCs under 980 nm laser excitation (Figure S11, Supporting Information). In addition to UC luminescence, there is also effective DS luminescence for the NCs. The absorption, photoluminescence excitation (PLE), and photoluminescence (PL) spectra of Cs_2_NaYb_1‐x_Er_x_Cl_6_ (x = 0, 0.03, 0.06, 0.09, and 0.12) NCs were recorded. The absorption spectra of Er^3+^ doped NCs not only include the matrix's absorption of ultraviolet and near‐infrared light but also the weak intrinsic absorption of Er^3+^ (Figure S12a,b, Supporting Information). The PLE spectra monitored at 445 and 1542 nm show the same stimulating components (Figure S13a,b, Supporting Information), which confirms that the intrinsic emission of Er^3+^ ions originate from energy transfer from STEs of the host NCs to Er^3+^ ions. The DS emission intensity initially increases, approaches the strongest, and then decreases (Figure S14a,b, Supporting Information). The PL lifetimes of STE emission conform to this variation pattern (Figure S15, Supporting Information). The initial increase of STEs emission intensity can be attributed to the enhanced electron‐phonon coupling effect caused by Jahn‐Teller distortion of the [YbCl_6_]^3−^ octahedron through the doping of Er^3+^ ions,^[^ ^15^ ^]^ while the subsequent decrease of STEs emission intensity with higher doping concentration is due to the improved energy transfer from STEs to doping ions. The blue emission band is STE emission, while NIR emission originates from ^4^I_13/2_→^4^I_15/2_ transition of Er^3+^ ions (Figure 2b). The optimal PLQY is 35.88% (Figure S16, Supporting Information). Notably, the blue emission band is STE emission, rather than that of OA and OAL, which is because the emission intensity of Cs_2_NaYb_0.94_Er_0.06_Cl_6_ NCs is much higher than that of OA and OLA, and their emission peak positions and PL lifetimes are also different (Figure S17a,b, Supporting Information). And the mechanism of DS PL is shown in Figure S18 (Supporting Information). Interestingly, as the temperature increases, although the emission intensity of DS emission decreases, the emission wavelength undergoes a significant redshift after 330 K (Figure S19a,b, Supporting Information; insets in Figure 2c), which is because as the temperature increases, the number of phonons in the lattice increases, and the interaction between excitons and phonons strengthens, resulting in a decrease in the excited state energy.^[^ ^16^ ^]^ Evidently, the DS luminescent color changes from blue to cyan to yellow as the temperature increases from 330 to 430 K (Figure 2c). It should be noted that the temperature in experiment about temperature‐dependent multi‐color emissions can only reach a maximum of 430 K, and cannot reach higher temperatures because when the temperature exceeds 430 K, its luminescent color is basically invisible to the naked eye (Figures S19a, S20, Supporting Information). More surprisingly, the Cs_2_NaYb_0.94_Er_0.06_Cl_6_ NCs demonstrate the ability of the reversible emission modal and color in response to water, as shown in Figure 2d. Before immersion in water, the NCs can emit UC green light and DS blue light, while they can only emit DS blue light after immersion in water (Figure S21, Supporting Information). This is because after soaking in water, the surface of the NCs will be slightly corroded (Figure S22, Supporting Information), resulting in defects increase and more non‐radiative processes. And after drying, the original dual‐mode and dual‐color characteristics of the NCs were restored (Figure S23, Supporting Information). But the NCs with the DS blue emission can be sustained well after being immersed in water for 180 days (Figure 2e; Figure S24, Supporting Information), although the surface of the NCs will be slightly corroded, resulting in a small amount of NaCl and CsCl, but still maintaining a high‐quality crystal structure (Figure S25, Supporting Information). And water‐soaked Cs_2_NaYb_0.94_Er_0.06_Cl_6_ NCs were dried, the XRD diffraction peak of NaCl and CsCl disappeared (Figure S26, Supporting Information). The NCs exhibit extraordinary water stability. The reason why the NCs have extraordinary water stability needs to be explained. First, the tolerance factor of Cs_2_NaYb_0.94_Er_0.06_Cl_6_ NCs is 0.92, and higher than other metal halide NCs (Table S3, Supporting Information), indicating the crystal structure is more stable. Second, lanthanide‐based metal halide NCs have higher ion dissociation and the hydrophobic effect of long‐chain ligands on the surface of the NCs.^[^ ^17^ ^]^ Surprisingly, Cs_2_NaYb_0.94_Er_0.06_Cl_6_ NCs have extremely superb environmental stability, which can be stored in the air for more than 18 months (Figure S27a–c, Supporting Information). Thermogravimetric analysis (TGA) shows the NCs that the decomposition temperature of nanocrystals is 243 °C, indicating good thermal stability (Figure S28, Supporting Information). Moreover, the luminescence spectra were continuously tested at 430 K for 60 min of Cs_2_NaYb_0.94_Er_0.06_Cl_6_ NCs, showing the luminescence intensity remained above 90% of its original value (Figure S29a, Supporting Information). And its XRD and Fourier transform infrared (FT‐IR) spectroscopy also indicate the NCs can maintain good stability at 430 K (Figure S29b,c, Supporting Information).

Cs_2_NaYb_1‐x_Er_x_Cl_6_ NCs not only exhibit static UC/DS dual‐mode emission under 980 nm laser/UV excitation but also dynamic PersL after the cessation of X‐ray irradiation. The cyan PersL of Cs_2_NaYb_0.94_Er_0.06_Cl_6_ NCs can be clearly recognized by the naked eye within 2 min at 77 and 300 K (Figure 2f–g). The contour plot of time‐dependent PersL spectra of Cs_2_NaYb_0.94_Er_0.06_Cl_6_ NCs after the cessation of X‐ray shows that PersL originates from STE recombination of the host and ^4^S_3/2_→^4^I_15/2_ and ^4^F_9/2_→^4^I_15/2_ transitions of Er^3+^ (Figure 2h). To understand the source of PersL generation, thermoluminescence (TL) spectrum of Cs_2_NaYb_0.94_Er_0.06_Cl_6_ NCs was measured (Figure 2i). By Gaussian fitting, five TL peaks were identified at 164, 175, 186, 225, and 447 K, indicating a continuous distribution of various types of PersL traps in Cs_2_NaYb_0.94_Er_0.06_Cl_6_ NCs. The trap depths (*E*) can be calculated using the Urbach formula:

(1) $$E=T_{m}\;/500$$

where *T_m_* is the temperature of the peak position for TL spectrum. The trap depths of Cs_2_NaYb_0.94_Er_0.06_Cl_6_ NCs were 0.33, 0.35, 0.37, 0.45, and 0.89 eV, respectively. Generally, the shallower trap of 0.33 eV is the main source to produce PersL, because a shallow trap depth is beneficial for the migration of carriers trapped in the defect to the emitting centers.^[^ ^18^ ^]^ To investigate whether the trap depth of the sample is affected by X‐ray radiation duration, the TL spectra of the sample irradiated for 10 s, 5 min, and 30 min showed the same curve (Figure S30, Supporting Information), indicating that there is no direct correlation between trap depth and X‐ray irradiation time. Moreover, PersL decay curve of Cs_2_NaYb_0.94_Er_0.06_Cl_6_ NCs was conducted by thermal stimulation (Figure S31, Supporting Information). After the PersL decayed for 225 s, the NCs were rapidly heated to 447 K, and PersL decay occurred again keeping at this temperature. The results show that two rounds of PersL decay curves are different, indicating that first and second PersL processes originate from trap sources at different depths. This also proves relatively deep traps exist in the NCs, and the electrons captured by these traps can escape to the conduction band by raising the temperature. Cs_2_NaYb_0.94_Er_0.06_Cl_6_ NCs exhibit high recyclability under X‐ray irradiation at 60 kV for 26 cycles (Figure 2j), which indicates that the NCs possess excellent resistance to X‐ray radiation damage. Moreover, the NCs possess the strong X‐ray absorption ability and ultra‐low phonon energy (Figure 2k; Figure S32, Supporting Information), which is beneficial for excellent PersL. To investigate the influence of irradiation time on PersL properties, the radioluminescence (RL) intensity under X‐ray irradiation of Cs_2_NaYb_0.94_Er_0.06_Cl_6_ NCs within 300 s and the PersL decay lifetimes under X‐ray irradiation within 5 min were measured (Figures S33, S34, Supporting Information). The result shows that the NCs should be irradiated with X‐ray for 5 min during the testing to ensure sufficient charging. Moreover, Cs_2_NaYb_1‐x_Er_x_Cl_6_ (x = 0.03, 0.06, 0.09, and 0.12) NCs can emit NIR light under X‐ray irradiation (Figure S35, Supporting Information).

**Figure 3:**
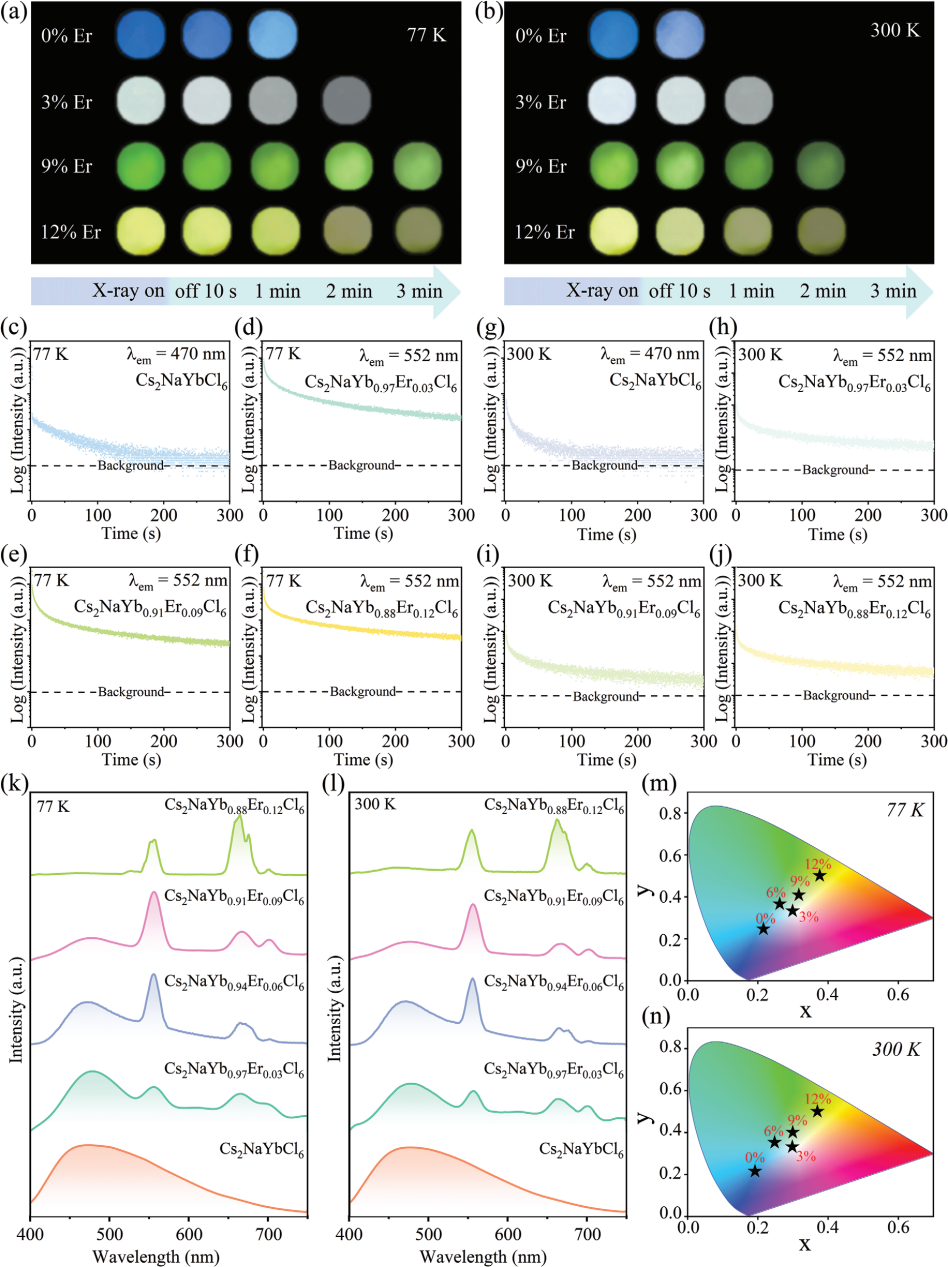
PersL photographs of Cs_2_NaYb_1‐x_Er_x_Cl_6_ (x = 0, 0.03, 0.09, and 0.12) NCs were captured after removing the X‐ray excitation source at a) 77 and b) 300 K. PersL decay curves of Cs_2_NaYb_1‐x_Er_x_Cl_6_ (x = 0, 0.03, 0.09, and 0.12) NCs were captured after removing the X‐ray excitation source at c–f) 77 and g–j) 300 K. k,l) PersL spectra of Cs_2_NaYb_1‐x_Er_x_Cl_6_ (x = 0, 0.03, 0.06, 0.09, and 0.12) NCs at 10 s after the cessation of X‐ray at 77 and 300 K. m,n) CIE chromaticity diagrams of Cs_2_NaYb_1‐x_Er_x_Cl_6_ (x = 0, 0.03, 0.06, 0.09, and 0.12) NCs were measured 10 s after the cessation of X‐ray at 77 and 300 K.

To further gain the color‐tunable PersL, the cross relaxation of Er^3+^ ions is cleverly utilized. In addition to the cyan PersL of Cs_2_NaYb_0.94_Er_0.06_Cl_6_ NCs, Cs_2_NaYbCl_6_, Cs_2_NaYb_0.97_Er_0.03_Cl_6_, Cs_2_NaYb_0.91_Er_0.09_Cl_6_, and Cs_2_NaYb_0.88_Er_0.12_Cl_6_ NCs all present blue, cool white, green, and yellow PersL at 77 and 300 K, respectively (**Figure** **3**a,b). The PersL decay curves of Cs_2_NaYb_1‐x_Er_x_Cl_6_ (x = 0, 0.03, 0.09, and 0.12) NCs at 77 K are shown as Figure 3c–f. The result shows that blue PersL can be clearly identified with the naked eye and last for at least 1 min, while cool white, green, and yellow PersL can be last for >2 min. Simultaneously, PersL decay curves of Cs_2_NaYb_1‐x_Er_x_Cl_6_ (x = 0, 0.03, 0.09, and 0.12) NCs at 300 K were also recorded (Figure 3g–j). PersL decay lifetime at 300 K is slightly shorter than at 77 K, which is consistent with the results of the material's TL spectrum. Figure 3k,l are PersL spectra of Cs_2_NaYb_1‐x_Er_x_Cl_6_ (x = 0, 0.03, 0.06, 0.09, and 0.12) NCs at 10 s after the cessation of X‐ray at 77 and 300 K, which are almost identical to their emission spectra under X‐ray radiation (Figure S36, Supporting Information), and show that the STE emission intensity decreases gradually with the increase of Er^3+^ doping concentration, which exhibits that the existence of effective energy transfer from STEs to Er^3+^ ions (Figure S37, Supporting Information). Meanwhile, the green emission of Er^3+^ ions has weakened, while their red emission has become stronger, which is because strong cross relaxation occurs when Er^3+^ doping concentration is enough high.^[^ ^19^ ^]^ The chromaticity coordinates of Cs_2_NaYb_1‐x_Er_x_Cl_6_ (x = 0, 0.03, 0.06, 0.09, and 0.12) NCs at 77 and 300 K show the PersL colors are blue, cool white, cyan, green and yellow (Figure 3m,n). Especially, the color temperature of cool white was calculated, and the value is ≈7232 K (details in the Supporting Information). Here, the highest doping concentration of Er^3+^ ions is only 12%, without achieving a higher concentration. This is because severe concentration quenching occurs when the concentration exceeds 12%, resulting in weak luminescence intensity that is invisible to the naked eye. (Figure S38a–c, Supporting Information). Moreover, Cs_2_NaYb_0.99_Tm_0.01_Cl_6_ and Cs_2_NaYb_0.99_Ho_0.01_Cl_6_ NCs also have PersL after removing the X‐ray excitation source, but their PersL color is not adjustable (Figure S39, Supporting Information).

**Figure 4:**
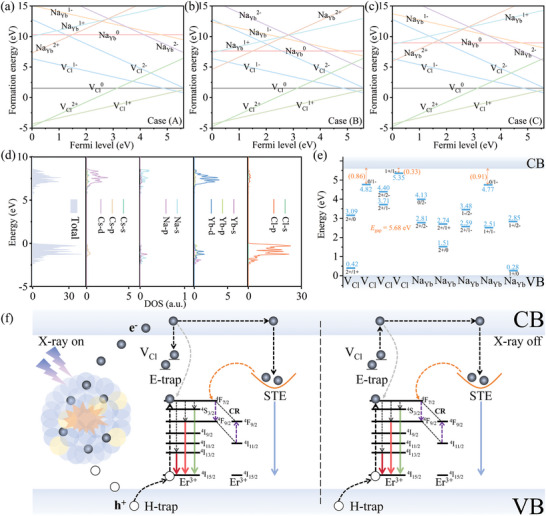
a–c) The formation energies of intrinsic point defects in various charge states, calculated as a function of the Fermi level within the band gap of Cs_2_NaYbCl_6_ NCs, considering three limiting cases (A–C) for atomic chemical potentials. d) The corresponding projected DOS of Cs_2_NaYbCl_6_ NCs. e) The thermodynamic charge transition levels of intrinsic point defects were calculated. The values in parentheses (in eV) represent the energy separations of levels relative to the host VB maximum (i.e., electron trap depth). E_gap_, energy gap. f) Schematic illustration of the proposed PersL mechanism.

To further gain insights into the origins of traps that play a key role in PersL, first‐principles calculations were performed within the frame of density functional theory (DFT). The formation energies of intrinsic point defects in various charge states as a function of the Fermi level in the band gap of Cs_2_NaYbCl_6_ NCs (**Figure** **4**a–c), considering three limiting cases (A–C) for the atomic chemical potentials (computational details in the Supporting Information). The electron trapping levels provided by chlorine vacancies (V_Cl_) are consistent in all three cases, while the electron trapping levels formed by small amount of Na^+^ replaces Yb^3+^ are the lowest in case B. The density of states (DOS) indicates a band gap of 5.68 eV, where the valence band is contributed by the Cl 3p state, and the conduction band is mainly composed of Yb 4d and Cs 4d orbitals (Figure 4d). Based on the formation energies of intrinsic point defects and band gap of Cs_2_NaYbCl_6_ NCs, the thermodynamic charge‐transition levels of the point defects are shown in Figure 4e. The results show that the chlorine vacancies (V_Cl_) provide electron trapping level *Ɛ*(1+/1−) with depth 0.33 eV, which is the trap source that provides efficient PersL. Moreover, the anti‐site Na_Yb_ electron trapping level *Ɛ*(1+/1−) with depth 0.91 eV and V_Cl_ trapping level *Ɛ*(0/1−) with depth 0.86 eV are appropriate for achieving effective thermally stimulated PersL at 447 K.

On the basis of the above discussion, the dominant mechanism of PersL was proposed in Figure 4f. Under X‐ray irradiation, free energetic electrons were obtained through the photoelectric effect. These electrons will collide with the atoms in the NCs produce other energetic electrons, and inject into conduction band. Part of the excited state electrons are captured by traps, while the other part relaxes to the STEs and high energy levels of Er^3+^. And then generates blue STE emission and intrinsic emission of Er^3+^. When the doping concentration of Er^3+^ is enough high, strong cross relaxation can occur, resulting in an increase in the red light and a decrease in the green light for Er^3+^, which is a key factor in achieving color tunability. After X‐ray irradiation is ceased, the electrons in shallow traps are released to the emitting center, thereby achieving multicolor PersL, which also relies on the process of cross‐relaxation.

**Figure 5:**
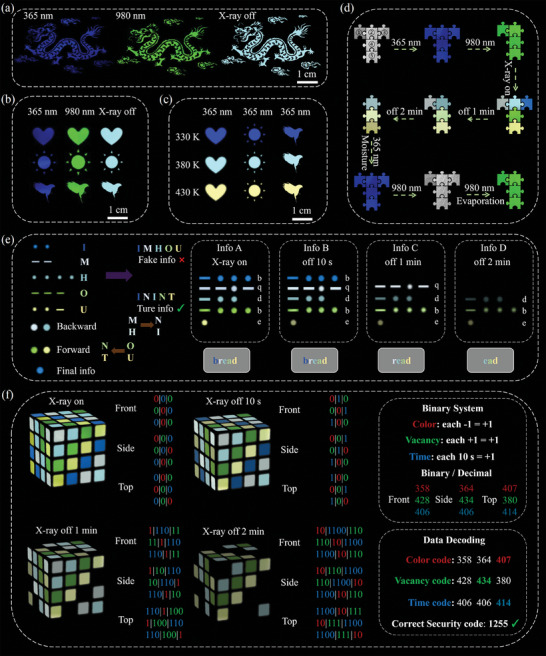
a) The “dragon” was coated on a black iron plate using Cs_2_NaYb_0.94_Er_0.06_Cl_6_ NCs, and it exhibited blue, green, and cyan emission colors under 365, 980 nm, and X‐ray excitation. b) The “heart,” “sun” and “eagle” shapes on a black iron plate, exhibiting blue, green, and cyan colors upon excitation at 365, 980 nm, and X‐ray, respectively. c) The “heart,” “sun” and “eagle” shapes on a black iron plate, exhibiting blue, cyan, and yellow emission colors upon 365 nm excitation at 330, 380, and 430 K, respectively. d) The letter “T” used in the process of digital information encryption is made from NCs (①Cs_2_NaYb_0.97_Er_0.03_Cl_6_, ②Cs_2_NaYb_0.94_Er_0.06_Cl_6_, ③Cs_2_NaYbCl_6_, ④Cs_2_NaYb_0.91_Er_0.09_Cl_6_, and ⑤Cs_2_NaYb_0.88_Er_0.12_Cl_6_) with different compositions. e) The encryption process of IMC is based on time‐division color multiplexing technology. f) Schematic illustration of the encryption and decryption process for binary and decimal cube codes.

Inspired by high encoding capacity of Er^3+^ doped Cs_2_NaYbCl_6_ NCs with multimode luminescence, anti‐counterfeiting patterns were designed. Without further processing, Cs_2_NaYb_0.94_Er_0.06_Cl_6_ NCs dispersed in n‐hexane to form luminescent ink. And then anti‐counterfeiting patterns were fabricated through screen printing, as shown in Figure S40 (Supporting Information). The patterns are invisible under sunlight. Under 365 nm UV excitation, the sophisticated “dragon” pattern emitted blue light. Upon 980 nm laser excitation, the “dragon” pattern exhibited a green color. After X‐ray irradiation ceased, the “dragon” pattern displayed cyan PersL (**Figure** **5**a). The “heart,” “sun” and “eagle” patterns were also printed on black iron plates and exhibited consistent luminescent behavior (Figure 5b). The luminescent ink is practically applied in a “100 RMB commemorative banknote,” and demonstrates high‐quality anti‐counterfeiting capabilities (Figure S41, Supporting Information). Additionally, the temperature‐dependent characteristics of the fluorescent ink support its use in anti‐counterfeiting applications (Figure 5c).

Given the rich luminescent modes of Cs_2_NaYb_1‐x_Er_x_Cl_6_ (x = 0, 0.03, 0.06, 0.09, and 0.12) NCs, information encryption applications have also been implemented. As shown in Figure 5d, a series of NCs (①Cs_2_NaYb_0.97_Er_0.03_Cl_6_, ②Cs_2_NaYb_0.94_Er_0.06_Cl_6_, ③Cs_2_NaYbCl_6_, ④Cs_2_NaYb_0.91_Er_0.09_Cl_6_, and ⑤Cs_2_NaYb_0.88_Er_0.12_Cl_6_) inks were coated on five non‐fluorescent cards and pieced together into the shape of “T” letter. After a series of actions (365 nm, 980 nm and X‐ray excitation, X‐ray erasing for 1 min and 2 min, 365 nm, 980 nm excitation after humidification, and 980 nm excitation after drying), corresponding letter (“T”) and number (“7” and “1”) transformations can be obtained.

By modulation of PersL lifetime and color for Cs_2_NaYb_1‐x_Er_x_Cl_6_ (x = 0, 0.03, 0.06, 0.09, and 0.12) NCs, the time‐division color multiplexing information encryption technology was implemented by International Morse Code (IMC). As presented in Figure 5e, when the X‐ray is turned off, Cs_2_NaYb_1‐x_Er_x_Cl_6_ (x = 0, 0.03, 0.06, 0.09, and 0.12) NCs, due to their differing persistence times, divide the channels into three distinct time intervals: 10 s, 1 min and 2 min. The operating rules are as follows: First, the five types of NCs are arranged into corresponding IMC to encode letters. Additionally, different PersL colors are assigned new meanings: when cool white and cyan PersL appear, the IMC shifts backward by one letter in the sequence. Similarly, when green and yellow PersL occurs, the IMC advances forward by one letter. When blue PersL appears, the IMC doesn't shift. So the corresponding encoding message is “bread.” After the X‐ray is turned off for 10 s, the read information displays message “bread.” 1 min after X‐ray cessation, the disappearance of blue PersL alters the message, leaving it as “read.” 2 min after X‐ray cessation, the read information is message “read.” At this point, the time‐division color multiplexing information encryption technology has been completed. Furthermore, more high‐level information encryption technology has been also performed. First, the five types of NCs were coated on the faces of magic cube to form the cube code (Figure S42, Supporting Information). As shown in Figure 5f, the security model functions through a mechanism that assigns specific attributes to each region's cube code: color disappearance (marked in red), number of cube vacancies (marked in green), and PersL duration code every 10 s (marked in blue). These attributes correspond to binary shifts of one bit (+1) in the respective regions. When excited by X‐ray, the cube code exhibits chaotic colors, representing the binary exchange algorithm “000.” After a delay of 10 s, all areas undergo the first transition, introducing binary ciphers “010,” “001” and “100.” 1 min later, the blue area disappearance and the cube develops several vacancy spaces, adjusting the binary ciphers to “111011,” “111110” and “110111.” After 2 min, the new binary ciphers “101100110,” “110101100” and “110010110” are formed. And then generate security codes “358, 364, 407, 428, 434, 380, 406, and 414” through binary‐to‐decimal conversion. Based on the algorithm, we established with “ color code (at 2 min) – vacancy code (at 2 min) – time code (at 2 min),” the final lock code is determined to be “1255.”

## 3 Conclusion

In summary, we have integrated five luminescent modes into a stable single‐host nanomaterial. Cs_2_NaYb_1‐x_Er_x_Cl_6_ (x = 0, 0.03, 0.06, 0.09, and 0.12) NCs display strong static and dynamic colorful luminescence in response to ultraviolet, 980‐nm laser and X‐ray. Additionally, the DS emission color has temperature dependence. Surprisingly, it also demonstrates the ability of the reversible emission modal and color in response to water. Theoretical calculations and experimental characterizations reveal the reason why the NCs can exhibit multi‐mode luminescence. And achieve outstanding high‐level anti‐counterfeiting and information encryption design and implementation. More importantly, the NCs have extremely excellent environmental stability and can be synthesized on a large scale, demonstrating promising commercial prospects. This work not only provides a new perspective for developing multi‐mode luminescent nanomaterials but also offers advanced methods for information encryption.

## Conflict of Interest

The authors declare no conflict of interest.

## Author Contributions

B.Y.K. and G.C.P. designed the experiments and explained the data. B.Y.K., G.C.P., and W.X. co‐authored the manuscript. B.Y.K. performed the experiments, measurements, characterizations, and data analyses. M.K.W. contributed to anti‐counterfeiting pattern design and PersL image acquisition. H.Y.T., Z.P.L., S.Y.S., and Y.X.L. contributed to PLQY measurement. W.W.Y. provided suggestions for manuscript. B.Y.K., G.C.P., W.X., and Y.L.M. discussed, reviewed, and provided feedback on the manuscript. All authors read and approved the final manuscript.

## Supporting information



Supporting Information

## Data Availability

The data that support the findings of this study are available in the supplementary material of this article.
