# Supplementary material for: Superior Multimodal Luminescence in a Stable Single‐Host Nanomaterial with Large‐Scale Synthesis for High‐Level Anti‐Counterfeiting and Encryption
Source: Adv Sci (Weinh). 2025 Jan 13;12(9):2415473. doi: 10.1002/advs.202415473 (PMC11884603; doi:10.1002/advs.202415473)
Supplement: Supplementary file 1 — Supporting Information [file ADVS-12-2415473-s001.pdf]

ADVANCED SCIENCE
Open Access

Supporting Information

for *Adv. Sci.*, DOI 10.1002/advs.202415473

Superior Multimodal Luminescence in a Stable Single-Host Nanomaterial with Large-Scale Synthesis for High-Level Anti-Counterfeiting and Encryption

Bingyin Kong, Gencai Pan, Mengke Wang, Hongye Tang, Zhipeng Lv, Shiyu Sun, Yuxin Luo, Wenwu You, Wen Xu* and Yanli Mao**

Supporting Information

Superior Multimodal Luminescence in a Stable Single-Host Nanomaterial with Large-Scale Synthesis for High-Level Anti-counterfeiting and Encryption

Bingyin Kong[1], Gencai Pan[1], Mengke Wang[1], Hongye Tang[1], Zhipeng Lv[1], Shiyu Sun[1], Yuxin Luo[1], Wenwu You[1], Wen Xu[2]* and Yanli Mao[1]**

[1]B. Kong, G. Pan, M. Wang, H. Tang, Z. Lv, S. Sun, Y. Luo, W. You, Y. Mao

Key Laboratory for High Efficiency Energy Conversion Science and Technology of Henan Province.

International Joint Research Laboratory of New Energy Materials and Devices of Henan Province, School of Physics and Electronics, Henan University, Kaifeng 475004, P. R. China.

[2]W. Xu

Key Laboratory of New Energy and Rare Earth Resource Utilization of State Ethnic Affairs Commission, School of Physics and Materials Engineering, Dalian Minzu University, Dalian 116600, P.R. China.

*E-mail: pangencai@126.com; xuwen@dlnu.edu.cn; ylmao@henu.edu.cn

Experimental Section:

Chemicals: Caesium carbonate (Cs_2CO_3, 99.9%, Aladdin); sodium acetate (NaAc, 99.99%, Aladdin); ytterbium acetate ($Yb(Ac)_3·4H_2O$, 99.9%, Aladdin); erbium acetate ($Er(Ac)_3·4H_2O$, 99.99%, Aladdin); chlorotrimethylsilane (TMS-Cl, technical grade 99%, Aladdin); 1-Octadecene (ODE, technical grade 90%, Aladdin); oleic acid (OA, technical grade AR, Sinopharm Group Chemical Reagent Co., LTD). oleylamine (OLA, technical grade 80%-90%, Aladdin); methyl acetate ($C_3H_6O_2$, technical grade AR, 98%, Aladdin); n-Hexane (C_6H_{14}, technical grade AR, Tianjin Fuyu Fine Chemical Co., LTD). All chemicals were used directly without purification.

Preparation of Cs-oleate (Cs-OA) stock solution: 5 mmol Cs_2CO_3 and 10 mL OA were loaded into a 50 mL three-neck flask. The mixture was heated under vacuum degassing to 120°C for 30 min until the Cs_2CO_3 was completely dissolved, followed by heating under a nitrogen atmosphere at 150°C for another 30 min. The resulting yellow transparent solution was then stored for future use.

Preparation of Na-oleatce (Na-OA) stock solution: 10 mmol NaAc and 10 mL OA were loaded into a 50 mL three-neck flask. The preparation procedure was the same as that used for preparing Cs-OA.

Synthesis of $Cs_2NaYbCl_6$ NCs: The synthesis of $Cs_2NaYbCl_6$ NCs employed a modified hot-injection technique. 1 mmol $Yb(Ac)_3$ was added to a mixture of 20 mL ODE, 5 mL OA, and 3 mL OLA in a 100 mL three-neck flask. In vacuum conditions, the mixture was placed in a constant temperature magnetic stirrer and heated to 120°C to remove moisture and oxygen. After complete dissolution of all precursors, it was continuously heated for 10 min under a nitrogen environment, gradually turning into a transparent light-yellow color. Subsequently, 2 mL of Cs-OA and 1 mL of Na-OA were swiftly injected into the mixture. After 5 min, the solution was heated to 190°C under a nitrogen atmosphere, and 1 mL of TMS-Cl was swiftly injected. After 1 min of violent reaction, the solution is stopped from heating and cooled with an ice bath. Subsequently, transfer the original solution to a centrifuge tube and centrifuge at 12000 rpm for 15 min. Discard the brown supernatant and disperse the resulting precipitate in n-hexane. Centrifuge at 12000 rpm for 10 min, repeating this process twice. Afterwards, centrifuge the solution at 2000 rpm for 30 s and discard the precipitate. $Cs_2NaYbCl_6$ NCs are dispersed in the supernatant.

Large-scale synthesis of $Cs_2NaYbCl_6$ NCs: The large-scale synthesis of $Cs_2NaYbCl_6$ NCs employed a modified hot-injection technique. 5 mmol $Yb(Ac)_3$ was added to a mixture of 100 mL ODE, 25 mL OA, and 15 mL OLA in a 300 mL three-neck flask. In vacuum conditions, the mixture was placed in a constant temperature magnetic stirrer and heated to 120°C to remove moisture and oxygen. After complete dissolution of all precursors, it was continuously heated for 10 min under a nitrogen environment, gradually turning into a transparent light-yellow color. Subsequently, 10 mL of Cs-OA and 5 mL of Na-OA were swiftly injected into the mixture. After 5 min, the solution was heated to 190°C under a nitrogen atmosphere, and 5 mL of TMS-Cl was swiftly injected. After 1 min of violent reaction, the solution is stopped from heating and cooled with an ice bath. Subsequently, transfer the original solution to a centrifuge tube, add an appropriate amount of methyl acetate, and centrifuge at 12000 rpm for 15 min. Discard the brown supernatant and disperse the resulting precipitate in n-hexane. Centrifuge at 12000 rpm for 10 min, repeating this process twice. Afterwards, centrifuge the solution at 2000 rpm for 30 s and discard the precipitate. $Cs_2NaYbCl_6$ NCs are dispersed in the supernatant.

Synthesis of $Cs_2NaYb_{1-x}Er_xCl_6$ (x = 0.03, 0.06, 0.09 and 0.12) NCs: The synthesis process of Er^{3+} doped $Cs_2NaYbCl_6$ NCs is highly analogous to the synthesis process of $Cs_2NaYbCl_6$ NCs discussed earlier. The only difference lies in the initial addition of $Er(Ac)_3$ into the three-neck flask, resulting in $Cs_2NaYb_{1-x}Er_xCl_6$ NCs with varying concentrations of x at 0.03, 0.06, 0.09 and 0.12 mmol. The quantities of other precursors and the preparation process remained unchanged, thereby yielding Er^{3+} doped $Cs_2NaYbCl_6$ NCs.

Large-scale synthesis of $Cs_2NaYb_{1-x}Er_xCl_6$ (x = 0.03, 0.06, 0.09 and 0.12) NCs: The large-scale synthesis process of Er^{3+} doped $Cs_2NaYbCl_6$ NCs is highly analogous to the large-scale synthesis process of $Cs_2NaYbCl_6$ NCs discussed earlier. The only difference lies in the initial addition of $Er(Ac)_3$ into the three-neck flask, resulting in $Cs_2NaYb_{1-x}Er_xCl_6$ NCs with different proportions of x at 0.03, 0.06, 0.09 and 0.12. The quantities of other precursors and the preparation process remained unchanged, thereby yielding Er^{3+} doped $Cs_2NaYbCl_6$ NCs.

Characterization: The 200 kV field emission transmission electron microscope (FETEM) (JEOL JEM-F200) was utilized for NCs morphology characterization. High-resolution transmission electron microscopy (HR-TEM), selected area electron diffraction (SAED), and Energy dispersive X-ray (EDX) were obtained by JEOL JEM-F200 instrument to determine the elemental composition and content of the material. Inductively Coupled Plasma Optical Emission Spectrometry (ICP-OES: Agilent 5110) analyses the specific content of the samples.

The NCs structure were analyzed and characterized using an X-ray diffractometer (DX-2700). The XPS Specturm was measured on the X-ray photoelectron spectrometer (AXIS SUPRA+). The UV-VIS-NIR absorption spectra of the samples were measured with an UV-VIS-NIR spectrophotometer (PE Lambda 950). Fourier transform infrared (FT-IR) spectra was measured on a Fourier Transform infrared spectrometer (VERTEX 70).

The FLS980 spectrometer (Edinburgh Instruments LTD., UK) was used to measure the fluorescence decay lifetime of the NCs. The excitation fluorescence curves of samples were measured using the FLS1000 spectrometer (Edinburgh Instruments LTD., UK). Using the FLS1000 spectrometer, both down-shifting fluorescence and up-conversion fluorescence were measured under excitation from a xenon lamp and an external 980 nm laser, respectively. The photoluminescence quantum yield (PLQY) of the samples were obtained using an integrating sphere attached to a fluorescence spectrometer (Edinburgh Instruments Ltd., UK). Subsequently, the PLQY values were calculated using the Edinburgh FLS1000 software package. Simultaneously, variable-temperature emission fluorescence spectra were measured at different temperatures.

Selecting Moxtek MAGPRO (70 kV 12 W) as the X-ray source for exciting samples. The radioluminescence (RL) spectra, Persistent Luminescence (PersL) decay curves, life decay curves and thermoluminescence (TL) curve were measured by FLS1000 combined with X-SASK-TCSS77-900K-3 equipment (temperature range: 77 to 800 K, accuracy: $\leq$0.1 K). A Sony Camera was used to capture fluorescence images of PersL under X-ray irradiation.

Stability: The samples were stored in deionized water for 6 months and in air at 24°C and 35% humidity for 18 months. We measured the photoluminescence (PL) spectra using FLS1000 and performed XRD measurements using DX-2700. Thermal gravimetric analysis (TGA) was performed using a "METTLER TOLEDO TGA/DSC3+" thermal analyzer.

Anti-counterfeiting: Take 10 g of NCs fully dispersed in n-hexane, and apply the mixed solution thoroughly onto a water-based screen-printed template. Position a black iron plate beneath the template and capture patterns under 365 nm lamps and 980 nm laser with a beam expander. Next, take another 10 g of nanocrystals and coat them completely onto an oil-based screen-printed template. Place either RMB or a black iron plate beneath the template and capture PersL patterns under the Moxtek MAGPRO (70 kV 12 W) and X-ray angiography system (SOMATOM Definition Flash, Siemens AG, Wittelsbacherplatz-2, DE-80333, Munich, Germany).

Calculation methods:

First-Principles Calculations:

All calculations in this study were performed with the Vienna ab initio Simulation Package (VASP) within the frame of density functional theory (DFT). The exchange correlation interactions of electron were described via the generalized gradient approximation (GGA) with PBE functional, and the projector augmented wave (PAW) method was used to describe the interactions of electron and ion. All calculations were based on the 319-atom unit cell of $Cs_2NaYbCl_6$ (space group $Fm\ \bar{3}m$). The Monkhorst-Pack scheme with a 1×1×1 k-point mesh was used for the integration in the irreducible Brillouin zone. The kinetic energy cutoff of 350 eV was chosen for the plane wave expansion. The lattice parameters and ionic position were fully relaxed, and the total energy was converged within 10^{-6} eV per formula unit. The final forces on all ions are less than 0.02 eV/Å.

For a specific defect α in charge state q, the formation enthalpy $\Delta H_f(\alpha, q)$ was calculated as follows:

$$\Delta H_f(\alpha,q) = E(\alpha,q) - E(perfect) - \sum n_i(E_i + \mu_i) + q\left[E_{VBM}(perfect) + E_f\right] + E_{corr} \quad (1)$$

Where $E(\alpha,q)$ and $E(perfect)$ are the total energies of the cell with the defect (α) in the charge q and the perfect host cell, respectively. E_f is the Fermi level referred to the VBM level ($E_{VBM}(perfect)$). n_i is the number of i atom added or removed, and μ_i is the chemical potential of the i atom. E_{corr} is the total correction for the formation enthalpy, including the band-filling correction, the potential alignment correction and the image charge correction. The defect formation energy depends on the chemical potentials of the constituent atoms. In the thermodynamic equilibrium, the chemical potentials are constrained within the following relation

$$2\mu_{Cs} + \mu_{Na} + \mu_{Yb} + 6\mu_{Cl} = \mu_{Cs_2NaYbCl_6} \quad (2)$$

Three limiting cases (case A-C) were considered, and the derived μ_i values therefrom were used to calculate formation enthalpy $\Delta H_f(\alpha, q)$, which represents the lower and upper limits for a point defect at a given Fermi-level under Cl-rich conditions.

The atomic chemical potentials of the other species were determined based on thermodynamic equilibrium conditions of various secondary phases containing these species. In this study, we consider three limiting cases (A-C):

Case A (the upper limits for $\mu_{Cs,Na}$ and the lower limit for μ_{Yb}:

$$\mu_{Cs} = \mu_{CsCl} - \mu_{Cl} \quad (3)$$

$$\mu_{Na} = \mu_{NaCl} - \mu_{Cl} \quad (4)$$

$$\mu_{Yb} = \mu_{Cs_2NaYbCl_6} - 2\mu_{Cs} - \mu_{Na} - 6\mu_{Cl} \quad (5)$$

Case B (the upper limits for $\mu_{Cs,Yb}$ and the lower limit for μ_{Na}:

$$\mu_{Cs} = \mu_{CsCl} - \mu_{Cl} \quad (6)$$

$$\mu_{Yb} = 1/3\left(\mu_{YbCl_3} - 3\mu_{Cl}\right) \quad (7)$$

$$\mu_{Na} = \mu_{Cs_2NaYbCl_6} - 2\mu_{Cs} - \mu_{Yb} - 6\mu_{Cl} \quad (8)$$

Case C (the upper limits for $\mu_{Na,Yb}$ and the lower limit for μ_{Cs}:

$$\mu_{Na} = \mu_{NaCl} - \mu_{Cl} \quad (9)$$

$$\mu_{Yb} = 1/3\left(\mu_{YbCl_3} - 3\mu_{Cl}\right) \quad (10)$$

$$\mu_{Cs} = 1/2\left(\mu_{Cs_2NaYbCl_6} - \mu_{Na} - \mu_{Yb} - 6\mu_{Cl}\right) \quad (11)$$

The total energies of the bulk materials CsCl, NaCl, and $YbCl_3$ were calculated to obtain the corresponding chemical potentials.

The thermodynamic charge-transition levels (or defect levels) within the band gap correspond to the Fermi-level position where a transition occurs from one charge state (q) to another (q'). The transition level $\varepsilon(q/q')$ with reference to the host VBM can be calculated via the equation below:

$$\varepsilon\left(\frac{q}{q'}\right) = \frac{\Delta H_f(\alpha, q') - \Delta H_f(\alpha, q)}{q - q'} \quad (12)$$

The color temperature (Tc) calculation:

To calculate the color temperature (Tc), McCamy's approximation formula can be used. This formula estimates the Tc based on the (x, y) values from the CIE 1931 CC, especially for cases where the Tc is between 2000 K and 10000 K. The McCamy's approximation formula is as follows:

$$T_c = -449n^3 + 3525n^2 - 6823.3n + 5520.33 \quad (13)$$

$$n = \frac{x - x_e}{y - y_e} \quad (14)$$

Here, the variables xe and ye represent the CC of the equal energy white point. The standard coordinates for the equal energy white point are as follows:

$$x_e = 0.3320, y_e = 0.1858 \quad (15)$$

Substituting the CC of the cool white point into the McCamy's approximation formula yields a Tc of approximately 7232 K.

PL decay curves fitting and average life calculation:

The PL decay curves at 445, 552 and 665 nm were fitted by the formula:

$$I(t) = I_0 + A_1\, exp\left(\frac{-t}{\tau_1}\right) + A_2\, exp\left(\frac{-t}{\tau_2}\right) \quad (16)$$

Where, I_0 is the original fluorescence intensity, A_1 and A_2 are obtained by experimental results. τ_1 and τ_2 represent the fast and slow decay process, associated to quenching and direct radiative recombination of rare earth ions, respectively. The average lifetimes of all NCs with different Er^{3+} ions doping concentrations are calculated by the formula:

$$\tau = \frac{A_1\tau_1^2 + A_2\tau_2^2}{A_1\tau_1 + A_2\tau_2} \quad (17)$$

Table S1. The results ICP-OES measurements for $Cs_2NaYbCl_6$ and $Cs_2NaYb_{0.94}Er_{0.06}Cl_6$ NCs.

Sample	Element	Cs (mmol)	Na (mmol)	Yb (mmol)	Er (mmol)	Er/(Yb+Er) ×100%
$Cs_2NaYbCl_6$	Feed	2.00	1.00	1.00	0	0
	ICP	1.98	0.98	1.01	0	0
$Cs_2NaYb_{0.94}Er_{0.06}Cl_6$	Feed	2.00	1.00	0.94	0.06	6.00%
	ICP	1.99	0.99	0.99	0.06	5.71%

Table S2. Crystallographic Data of $Cs_2NaYbCl_6$ and $Cs_2NaYb_{0.94}Er_{0.06}Cl_6$ Derived from Rietveld Refinement of X-ray Diffraction Powder Diffraction Data.

Formula	$Cs_2NaYbCl_6$	$Cs_2NaYb_{0.94}Er_{0.06}Cl_6$
Crystal System	Cubic	Cubic
Space Group	$Fm\bar{3}m$ [225]	$Fm\bar{3}m$ [225]
Cell Length a/b/c (Å)	10.6896	10.6486
Cell Volume (Å^3)	1221.48	1207.48
Cell Angle (°)	90	90
Crystal Density (g/cm^3)	3.6326	3.6975
Temperature (K)	297	297
2θ (°)	20-80	20-80
Step Size (°)	0.01	0.01
Structure Refinement	Topas 5	Topas 5
Profile Function	PV_MOD	PV_MOD
R_{wp}	10.67%	11.12%
R_P	7.21%	7.81%
GOF	1.05	1.08
χ^2	1.11	1.17

Table S3. Comparison of tolerance factor of typical luminescent materials.

Formula	r_A (Å)	r_B (Å)	r_X (Å)	T
$Cs_2AgInCl_6$	1.67	0.98	1.81	0.88
$Cs_2NaInCl_6$	1.67	0.91	1.81	0.90
$Cs_2NaLaCl_6$	1.67	1.03	1.81	0.86
$Cs_2NaTbCl_6$	1.67	0.92	1.81	0.89
$Cs_2NaCeCl_6$	1.67	1.01	1.81	0.87
$Cs_2NaEuCl_6$	1.67	0.95	1.81	0.88
$CsPbCl_3$	1.67	1.19	1.81	0.82
$CsPbBr_3$	1.67	1.19	1.96	0.81
$Cs_2NaYb_{0.94}Er_{0.06}Cl_6$	1.67	0.87	1.81	0.92

In 1926, Goldschmidt formulated the tolerance factor concept for perovskites; the relationship between the ionic radii of the A-site cation, B-site cation and X-site anion obtained by applying simple trigonometry is given in equation [1] (15):

$$T = \frac{r_A + r_B}{\sqrt{2}(r_A + r_X)} \quad (18)$$

with r_A being the ionic radii of the A-site cation, r_B the ionic radii of the B-site cation and r_X the ionic radii of the X-site anion, respectively.

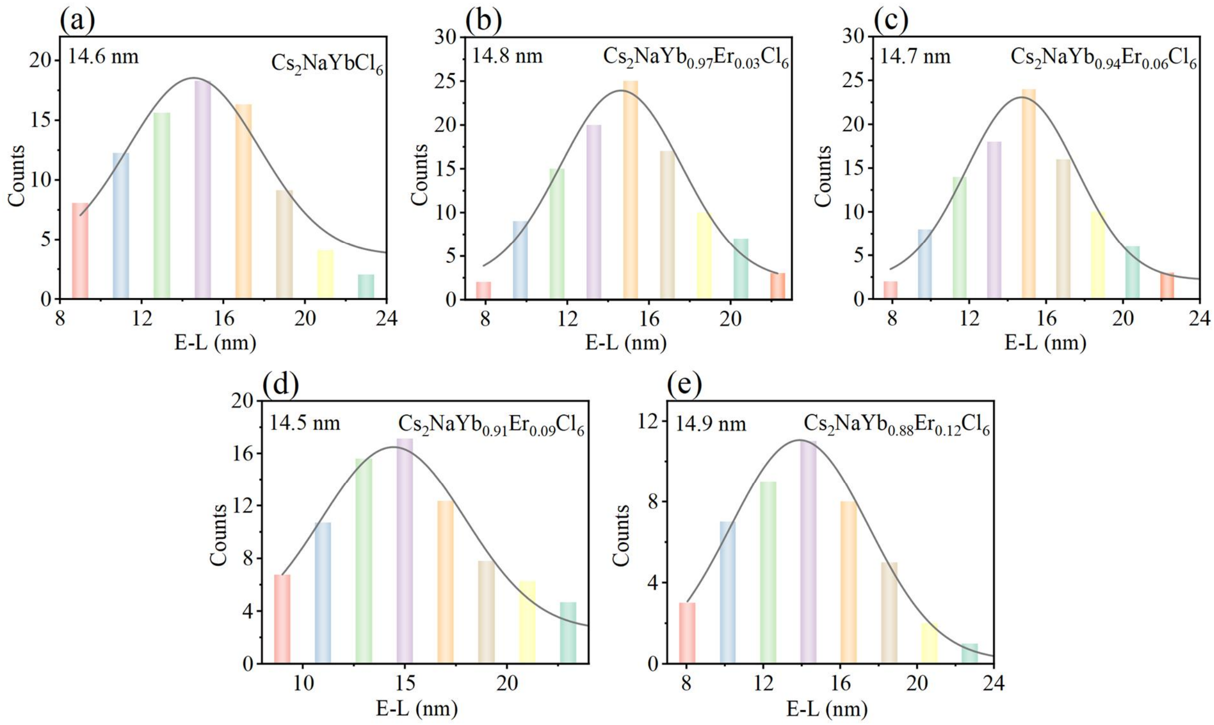

Figure S1. a-e) Size distribution diagram of $Cs_2NaYb_{1-x}Er_xCl_6$ NCs.

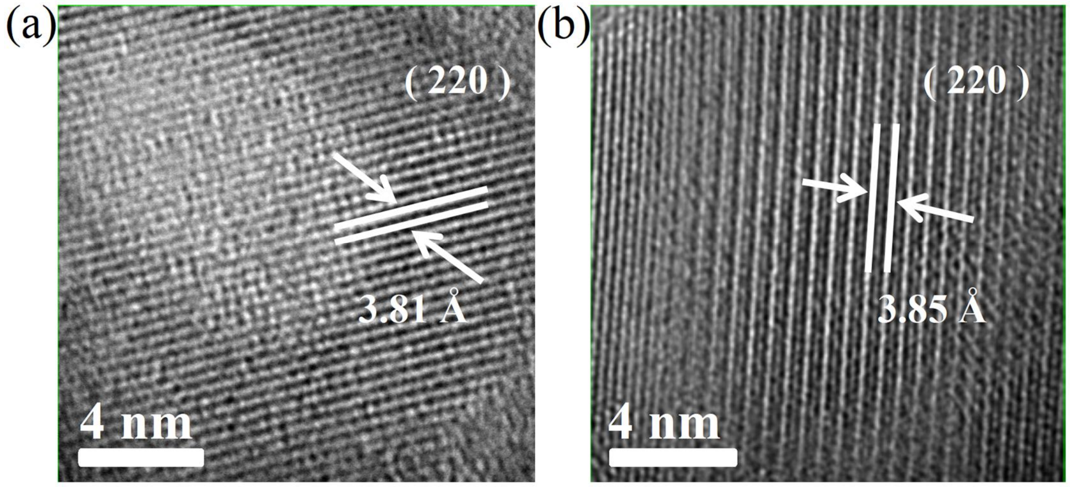

Figure S2. a-b) HR-TEM images of $Cs_2NaYbCl_6$ and $Cs_2NaYb_{0.94}Er_{0.06}Cl_6$ NCs.

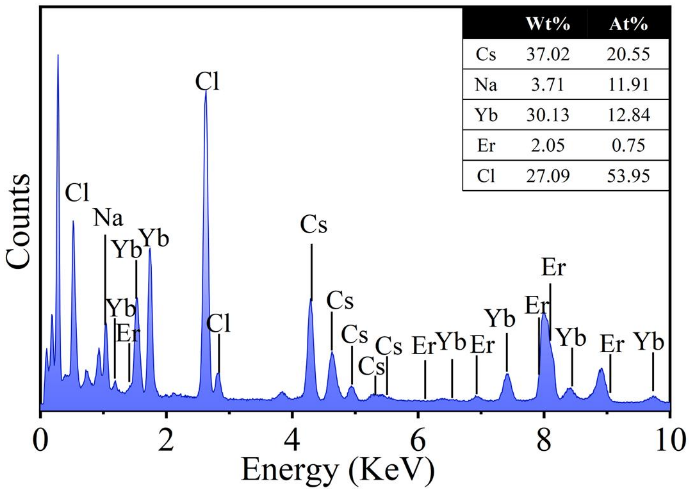

Figure S3. The EDX spectrum of $Cs_2NaYb_{0.94}Er_{0.06}Cl_6$ NCs.

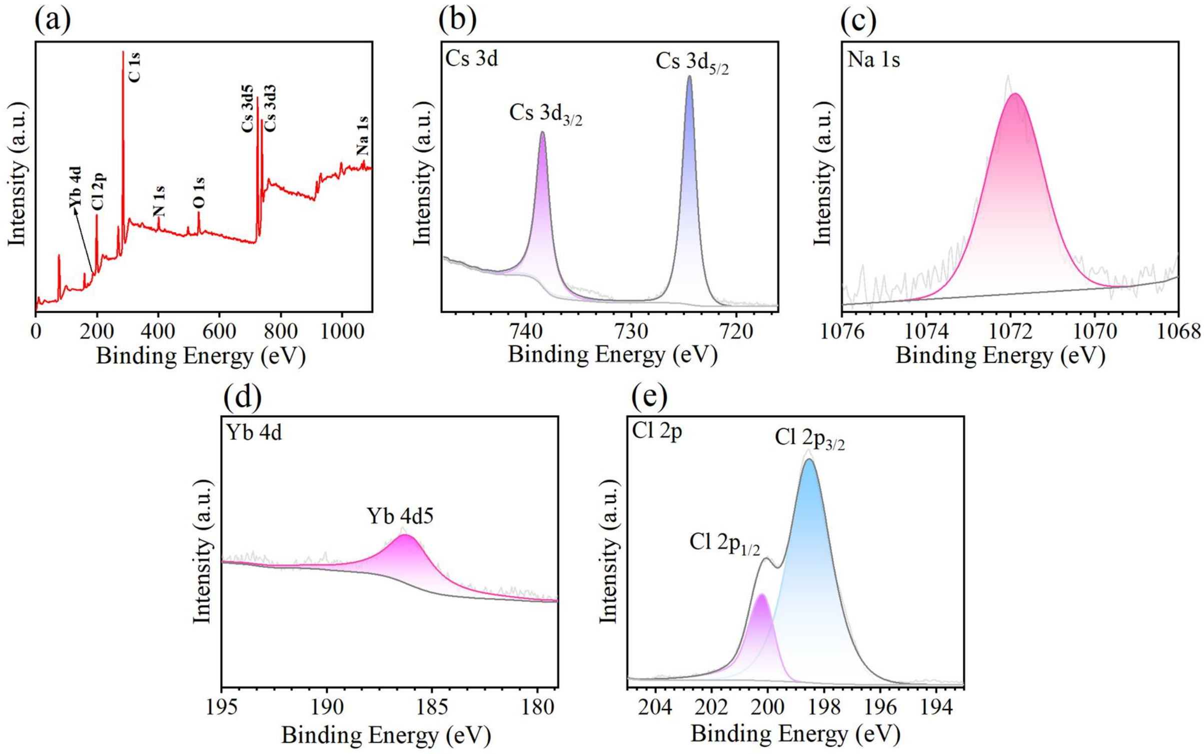

Figure S4. a) The XPS spectra of $Cs_2NaYbCl_6$ NCs. b–e) The corresponding high-resolution XPS spectra of Cs (3d), Na (1s), Yb (4d) and Cl (2p).

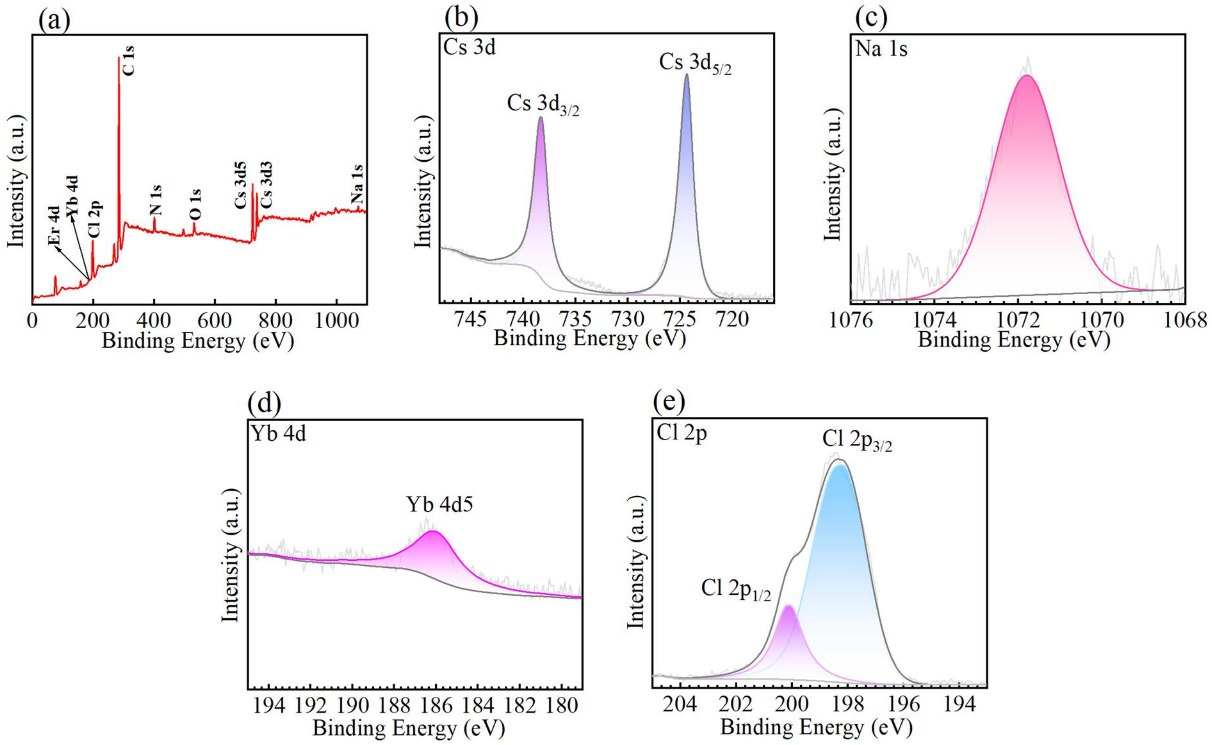

Figure S5. a) The XPS spectra of $Cs_2NaYb_{0.94}Er_{0.06}Cl_6$ NCs. b–e) The corresponding high-resolution XPS spectra of Cs (3d), Na (1s), Yb (4d) and Cl (2p).

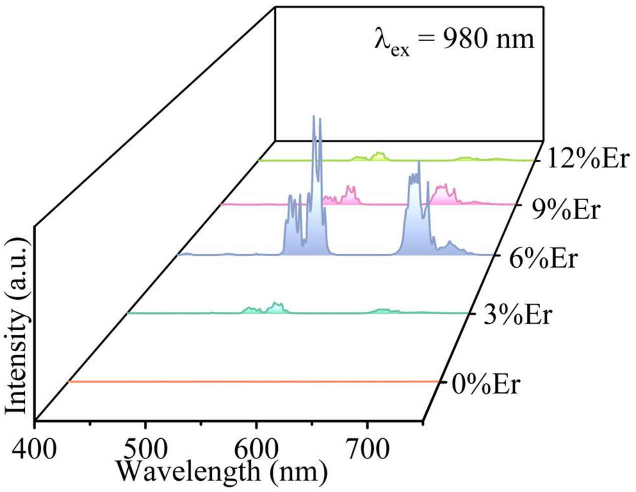

Figure S6. UC emission PL spectra of $Cs_2NaYb_{1-x}Er_xCl_6$ NCs under excitation of 980 nm laser.

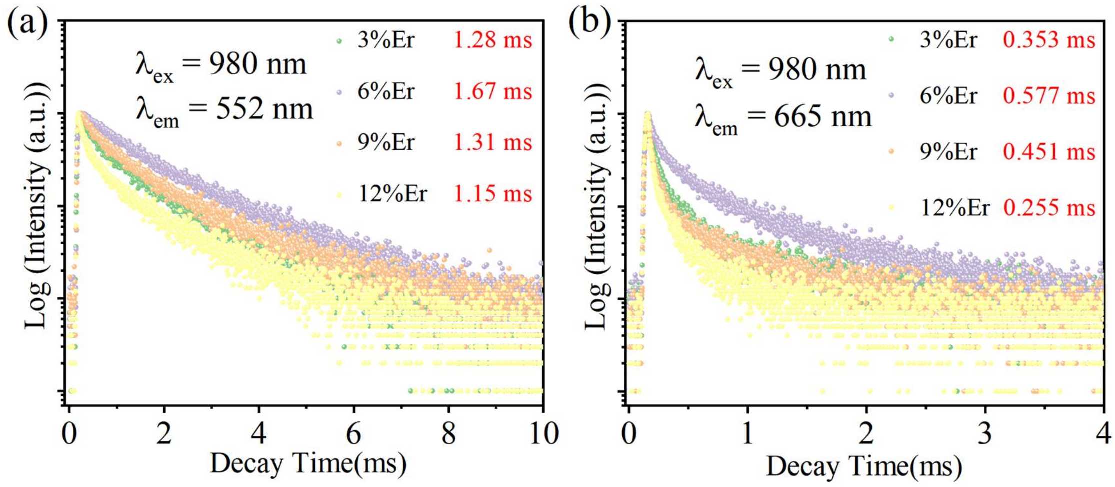

Figure S7. PL lifetime decay curves of $Cs_2NaYb_{1-x}Er_xCl_6$ NCs. a) at 552 nm and b) 665 nm excited by 980 nm laser.

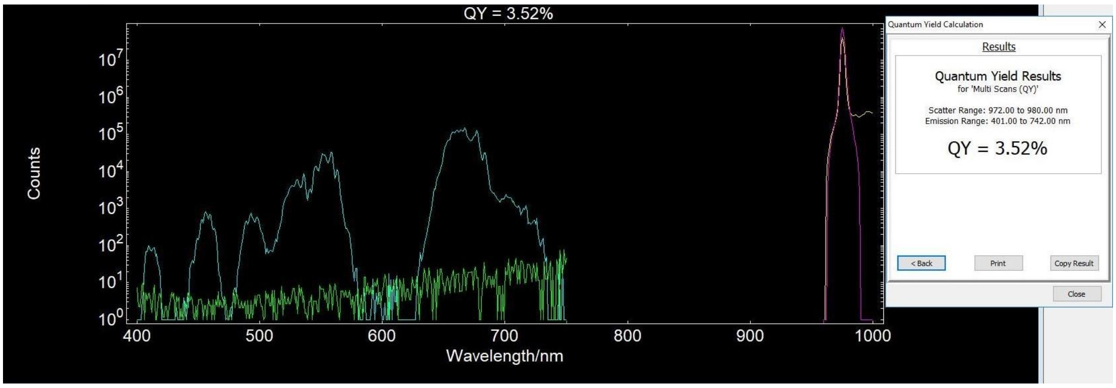

Figure S8. The UC PLQY of $Cs_2NaYb_{0.94}Er_{0.06}Cl_6$ NCs.

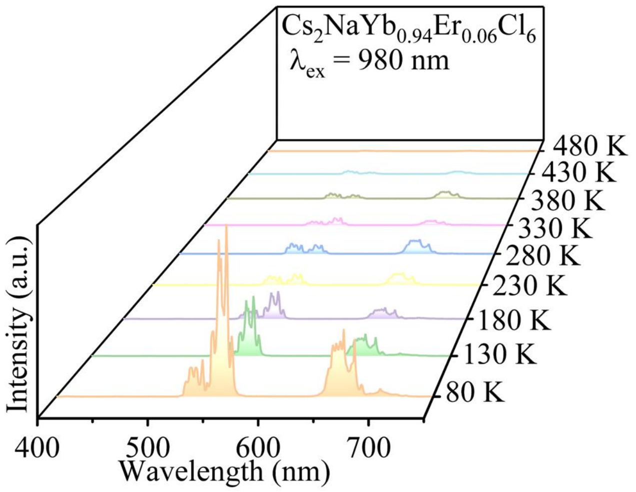

Figure S9. UC variable temperature spectrum of $Cs_2NaYb_{0.94}Er_{0.06}Cl_6$ NCs.

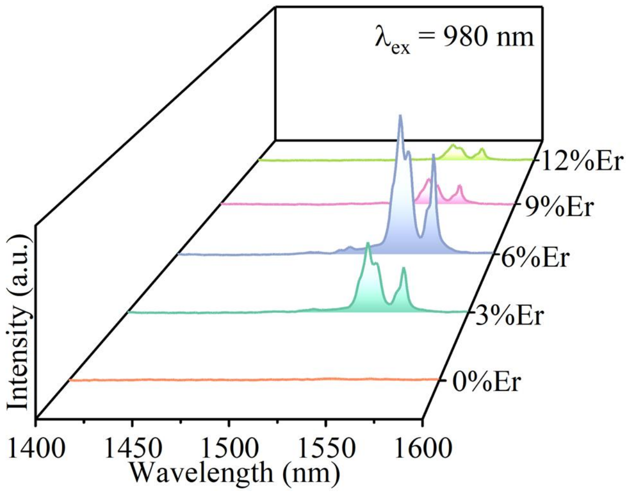

Figure S10. NIR photoluminescence spectra of $Cs_2NaYb_{1-x}Er_xCl_6$ (x = 0, 0.03, 0.06, 0.09, 0.12 mmol) NCs under excitation of 980 nm laser.

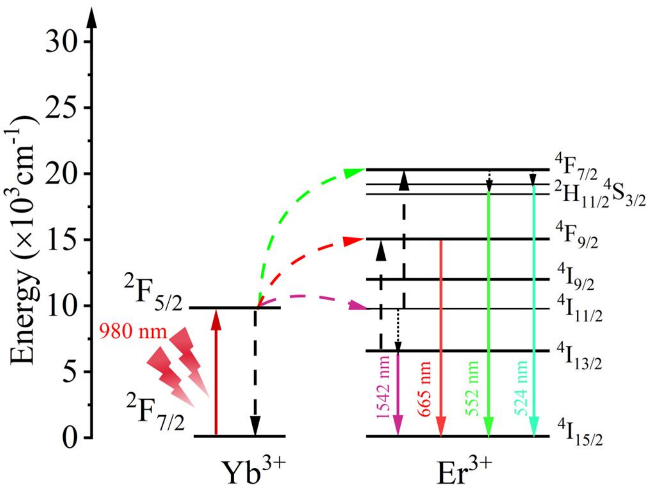

Figure S11. Proposed luminescence mechanism diagram under 980 nm.

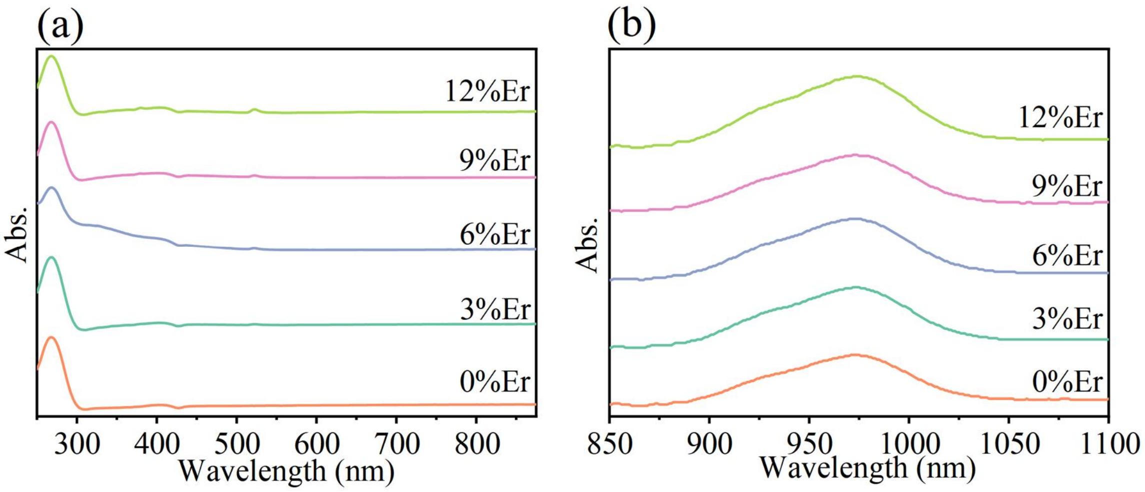

Figure S12. a) Ultraviolet absorption spectra and b) Infrared absorption spectra of $Cs_2NaYb_{1-x}Er_xCl_6$ NCs.

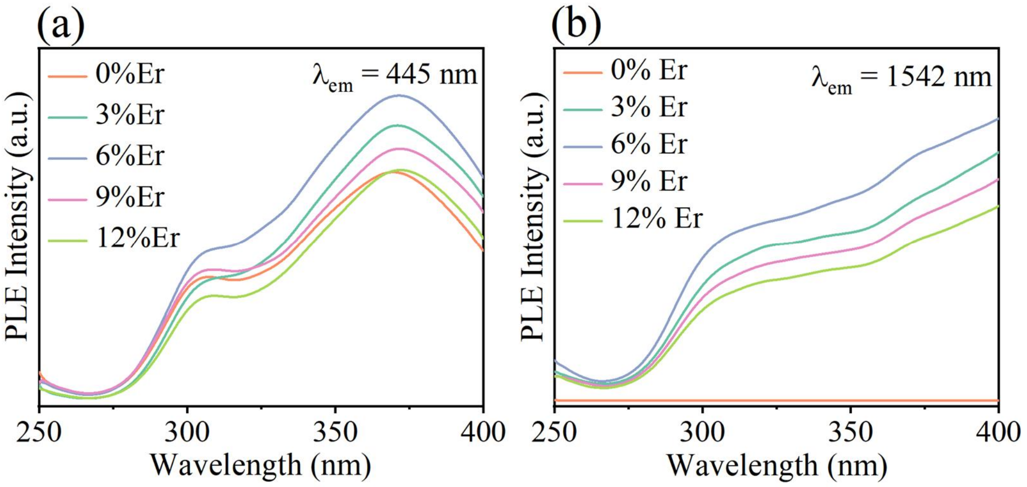

Figure S13. PLE spectra at 445 nm and 1542 nm of $Cs_2NaYb_{1-x}Er_xCl_6$ NCs.

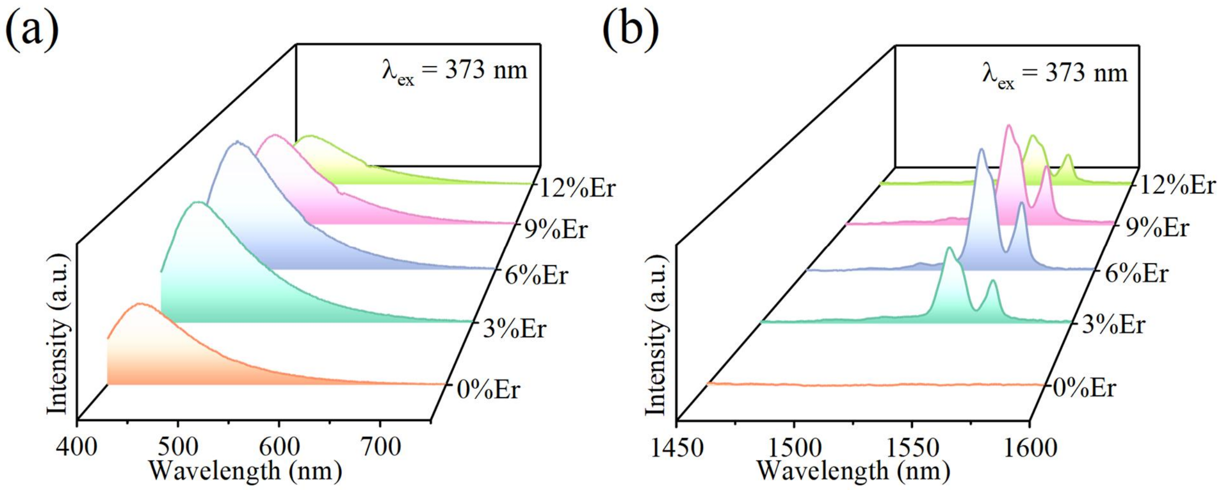

Figure S14. a) Visible PL and b) NIR PL spectra of $Cs_2NaYb_{1-x}Er_xCl_6$ NCs.

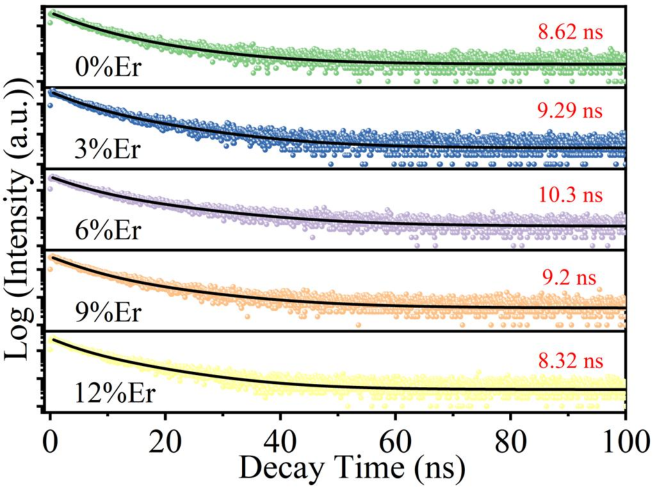

Figure S15. PL lifetime decay curve of $Cs_2NaYb_{1-x}Er_xCl_6$ NCs was monitored at 445 nm.

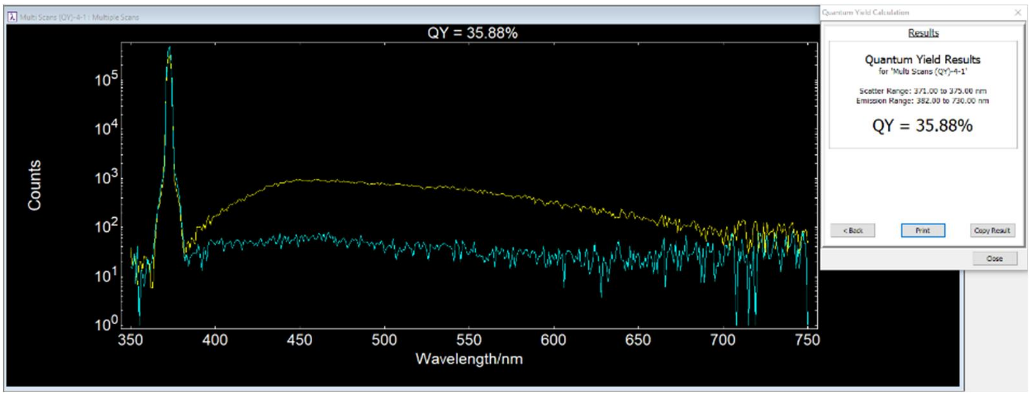

Figure S16. PLQY of $Cs_2NaYb_{0.94}Er_{0.06}Cl_6$ NCs.

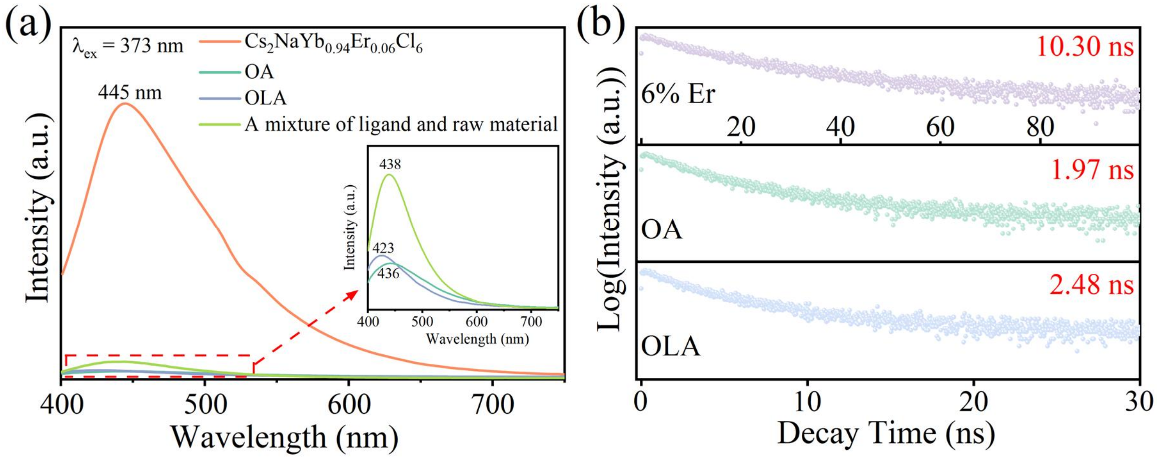

Figure S17. a) PL spectra of OA, OLA, and $Cs_2NaYb_{0.94}Er_{0.06}Cl_6$ NCs. **b)** PL lifetime decay curve of OA, OLA and $Cs_2NaYb_{0.94}Er_{0.06}Cl_6$ NCs.

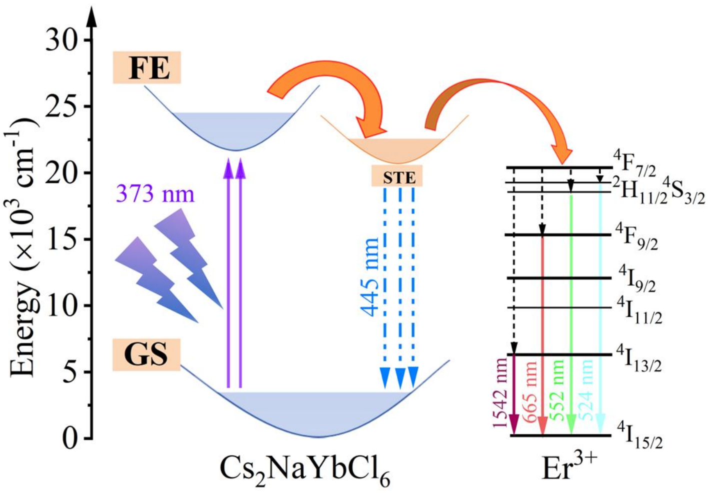

Figure S18. Proposed luminescence mechanism diagram under 373 nm.

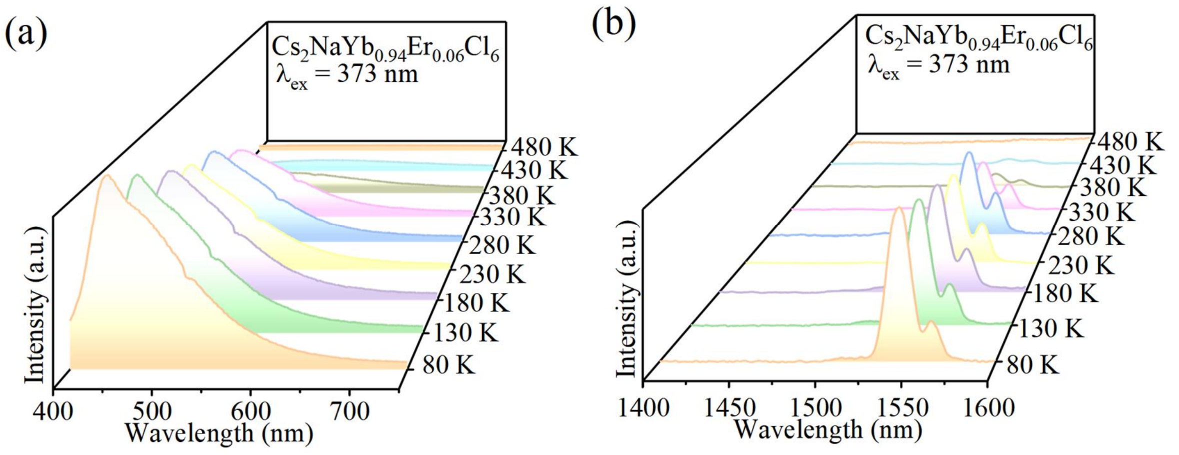

Figure S19. a) Visible variable temperature spectrum and b) NIR variable temperature spectrum of $Cs_2NaYb_{0.94}Er_{0.06}Cl_6$ NCs.

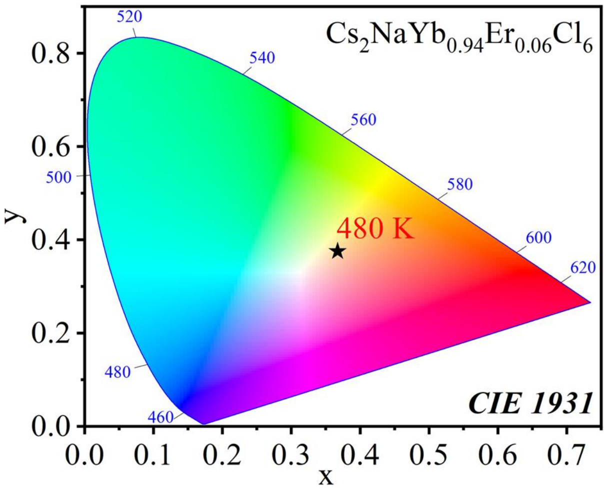

Figure S20. CIE chromaticity diagrams of $Cs_2NaYb_{0.94}Er_{0.06}Cl_6$ NCs at 480 K.

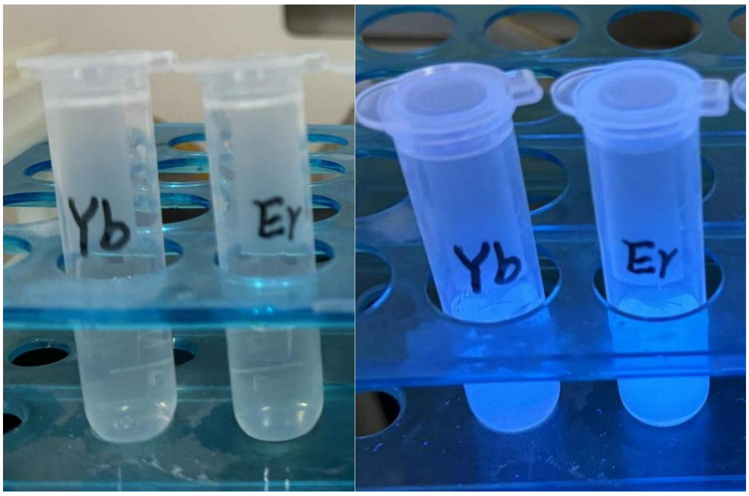

Figure S21. The left image shows the appearance of $Cs_2NaYbCl_6$ and $Cs_2NaYb_{0.94}Er_{0.06}Cl_6$ NCs dissolved in water under daylight illumination, while the right image displays their appearance under 365 nm UV light.

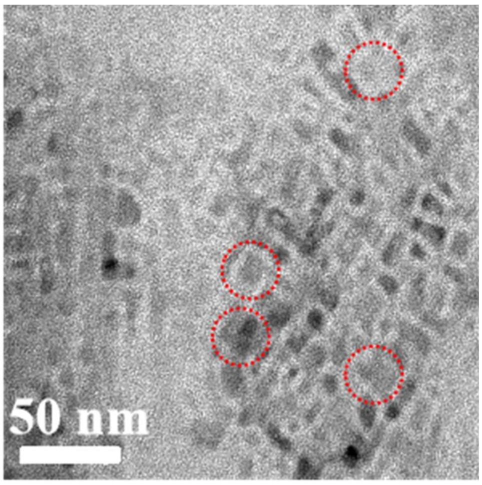

Figure S22. The TEM of $Cs_2NaYb_{0.94}Er_{0.06}Cl_6$ NCs after water stability testing.

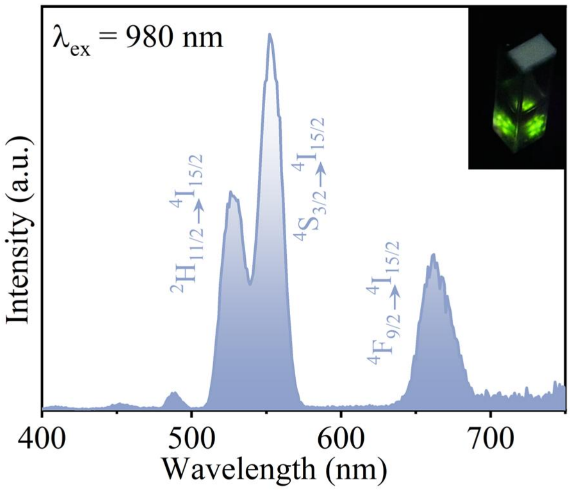

Figure S23. PL spectra of heat-treated $Cs_2NaYb_{0.94}Er_{0.06}Cl_6$ NCs under 980 nm excitation. The inset in the upper right corner shows an image of the sample under 980 nm excitation.

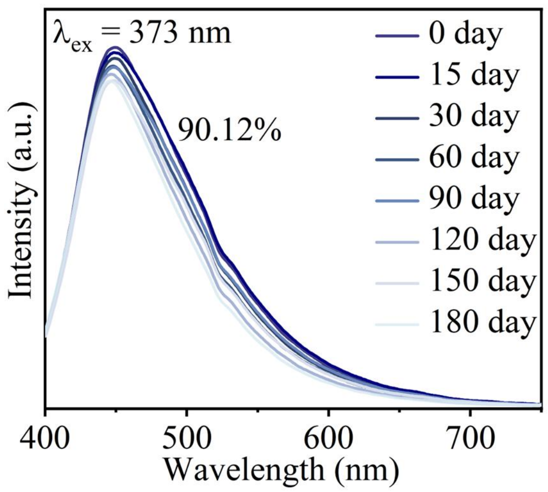

Figure S24. Emission spectra of $Cs_2NaYb_{0.94}Er_{0.06}Cl_6$ NCs in aqueous solution at different time intervals.

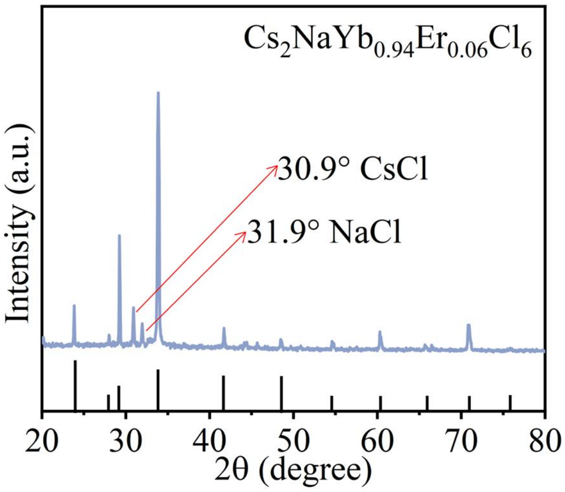

Figure S25. XRD data of $Cs_2NaYb_{0.94}Er_{0.06}Cl_6$ NCs after water stability testing.

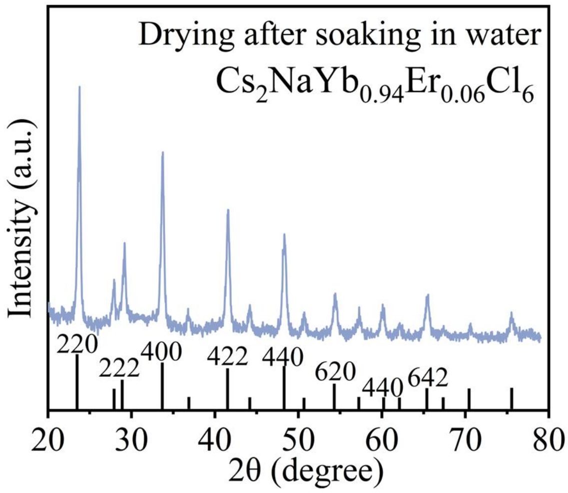

Figure S26. XRD pattern of water soaked $Cs_2NaYb_{0.94}Er_{0.06}Cl_6$ NCs after drying.

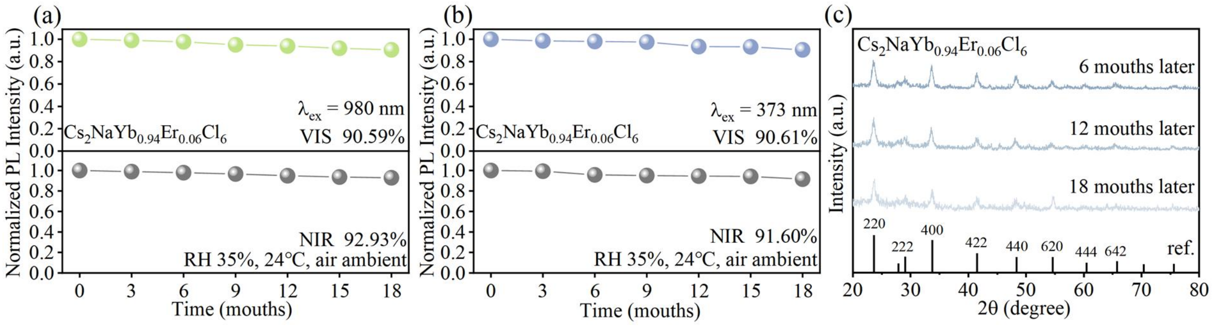

Figure S27. Normalized luminescence intensity statistics of $Cs_2NaYb_{0.94}Er_{0.06}Cl_6$ NCs under a) 373 nm and b) 980 nm light excitation (about humidity of 35% and 24°C). c) Comparison of XRD patterns of $Cs_2NaYb_{0.94}Er_{0.06}Cl_6$ NCs after being stored in air (at 24°C and 35% humidity) for 6 months, 12 months, and 18 months.

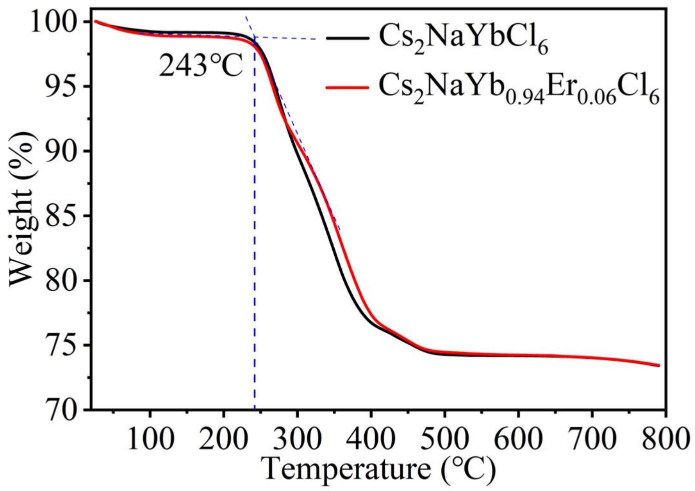

Figure S28. TGA graphs of $Cs_2NaYbCl_6$ and $Cs_2NaYb_{0.94}Er_{0.06}Cl_6$ NCs.

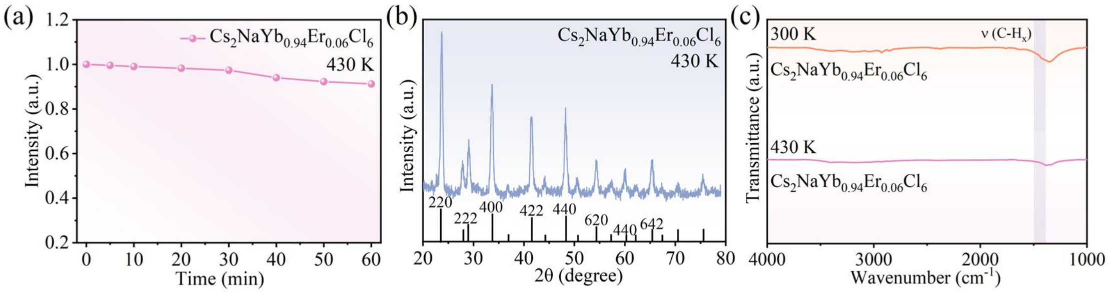

Figure S29. a) The luminescence spectra were continuously tested at 430 K for 60 min of $Cs_2NaYb_{0.94}Er_{0.06}Cl_6$ NCs. **b)** XRD data of $Cs_2NaYb_{0.94}Er_{0.06}Cl_6$ NCs after heating at 430 K. **c)** FT-IR spectra of $Cs_2NaYb_{0.94}Er_{0.06}Cl_6$ NCs at 300 K and 430 K.

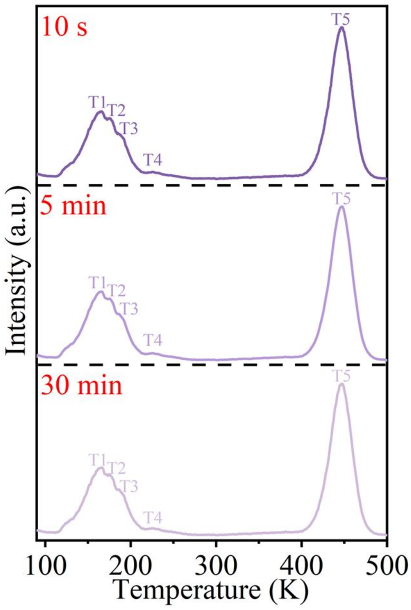

Figure S30. TL curves of $Cs_2NaYb_{0.94}Er_{0.06}Cl_6$ NCs were recorded following X-ray irradiation for 10 s, 5 min, and 30 min, respectively.

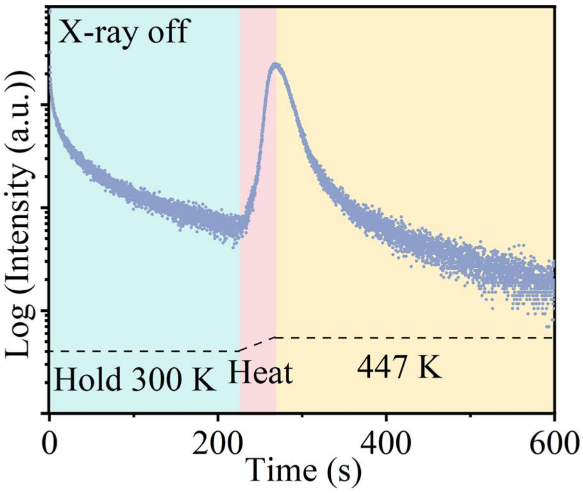

Figure S31. PersL decay curve of $Cs_2NaYb_{0.94}Er_{0.06}Cl_6$ NCs conducted by thermal stimulation (the sample was continuously irradiated by X-ray of 60 kV for 30 min, then the X-ray was ceased to obtain the PersL decay curve, and subsequently the sample was quickly heated to 447 K after 225 s).

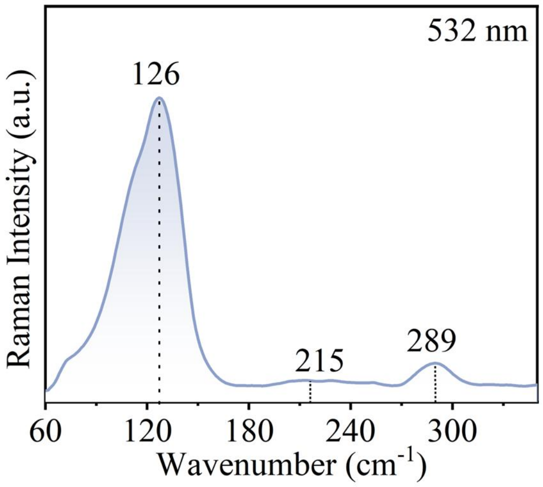

Figure S32. Raman spectrum of $Cs_2NaYb_{0.94}Er_{0.06}Cl_6$ NCs under 532 nm laser.

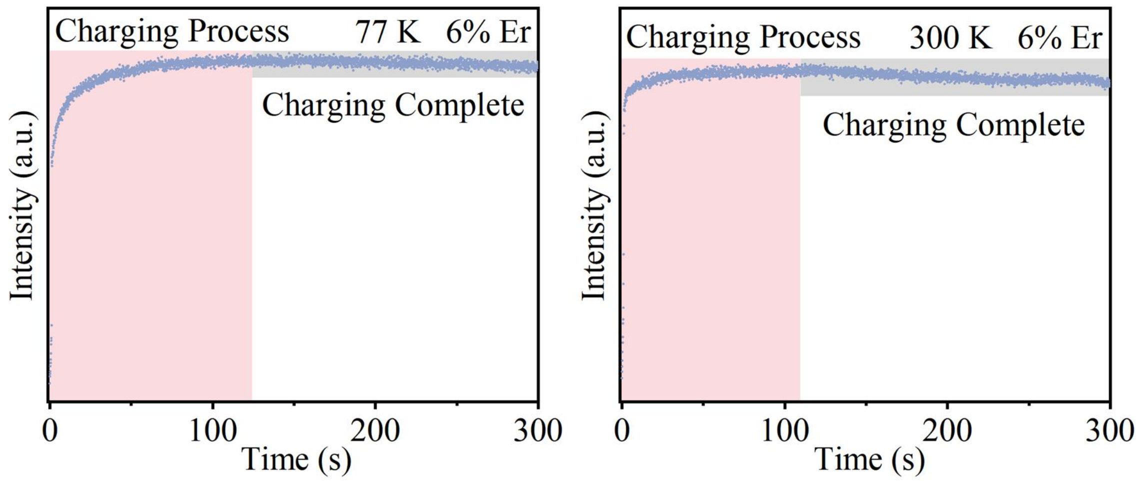

Figure S33. The Change in RL intensity of $Cs_2NaYb_{0.94}Er_{0.06}Cl_6$ NCs within 300 s at 77 K and 300 K.

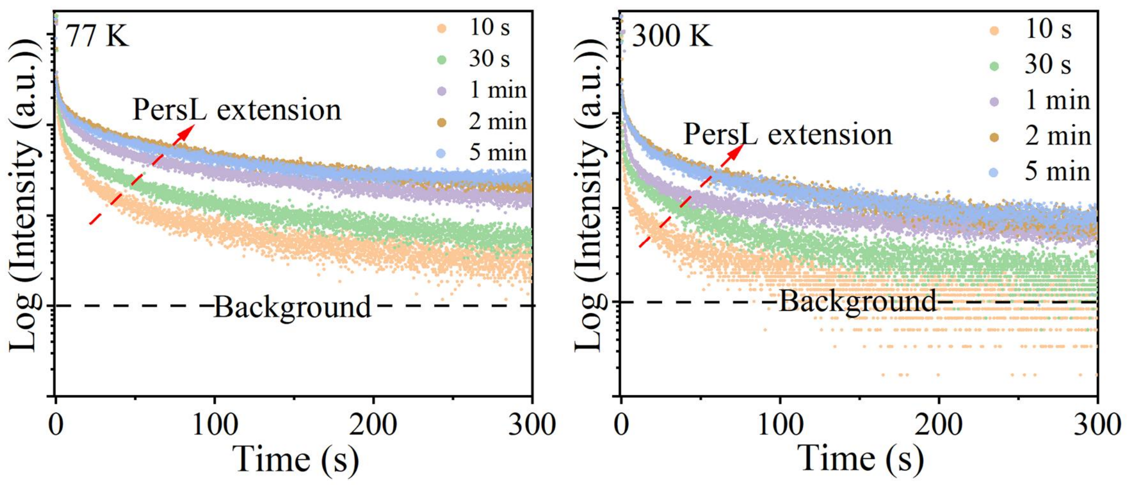

Figure S34. The PersL decay curves of $Cs_2NaYb_{0.94}Er_{0.06}Cl_6$ NCs as a function of X-ray irradiation time at 77 K and 300 K.

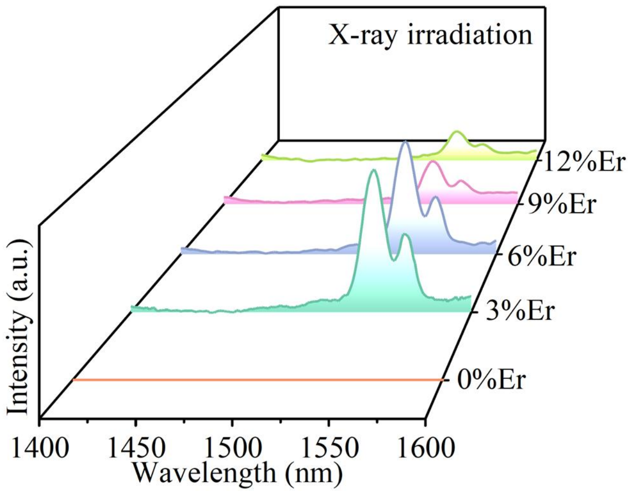

Figure S35. X-ray radioluminescence NIR spectra of $Cs_2NaYb_{1-x}Er_xCl_6$ (x = 0, 0.03, 0.06, 0.09, 0.12 mmol) NCs.

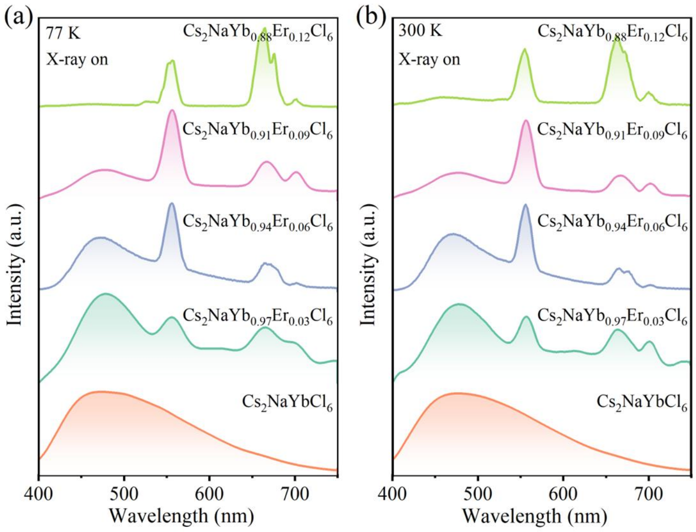

Figure S36. a-b) The emission spectrum under X-ray radiation of $Cs_2NaYb_{1-x}Er_xCl_6$ (x = 0, 0.03, 0.06, 0.09 and 0.12) NCs at 77 and 300 K.

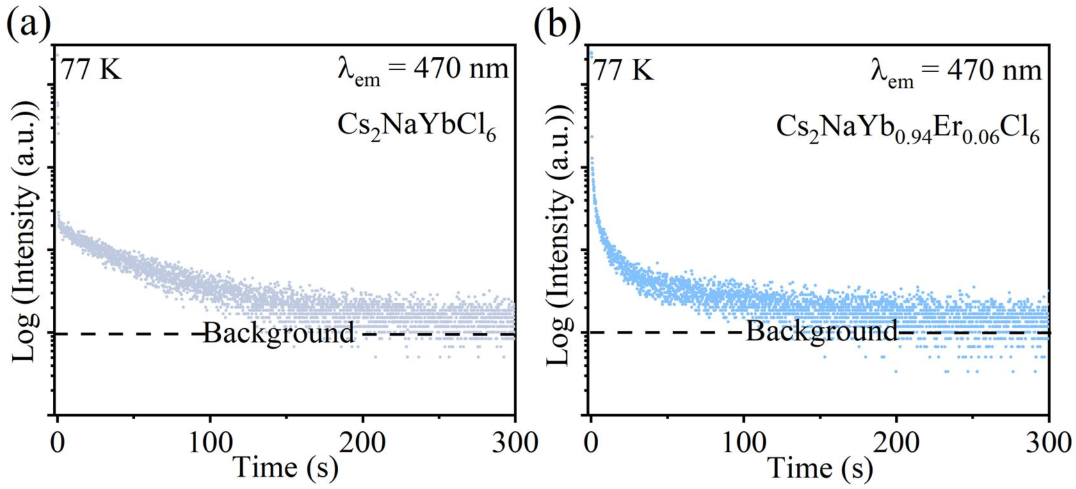

Figure S37. After removing the X-ray excitation source at 77 K, the PersL decay curves of a) $Cs_2NaYbCl_6$ and b) $Cs_2NaYb_{0.94}Er_{0.06}Cl_6$ NCs is obtained by monitoring the emission position at 470 nm.

The energy transfer efficiency η_T from STEs to Er^{3+} was determined by the following formula:

$$\eta_T = 1 - \frac{\tau_2}{\tau_1} \tag{19}$$

where τ_1 = 150 s and τ_2 = 110 s for the PersL lifetimes of the $Cs_2NaYbCl_6$ and $Cs_2NaYb_{0.94}Er_{0.06}Cl_6$ NCs, respectively. The energy transfer efficiency from STEs to Er^{3+} ions is up to about 27%.

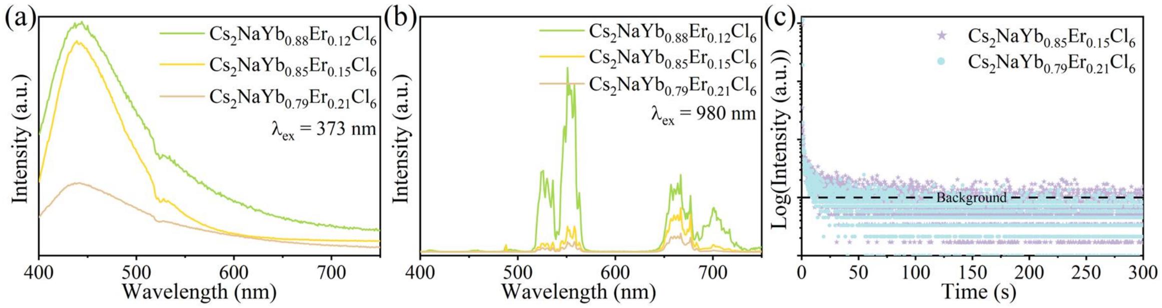

Figure S38 a) Visible PL spectra of $Cs_2NaYb_{1-x}Er_xCl_6$ (x = 0.12, 0.15, 0.21 mmol) NCs. **b)** UC emission PL spectra of $Cs_2NaYb_{1-x}Er_xCl_6$ (x = 0.12, 0.15, 0.21 mmol) NCs under excitation of 980 nm laser. **c)** PersL decay curves of $Cs_2NaYb_{1-x}Er_xCl_6$ (x = 0.15, 0.21 mmol) NCs.

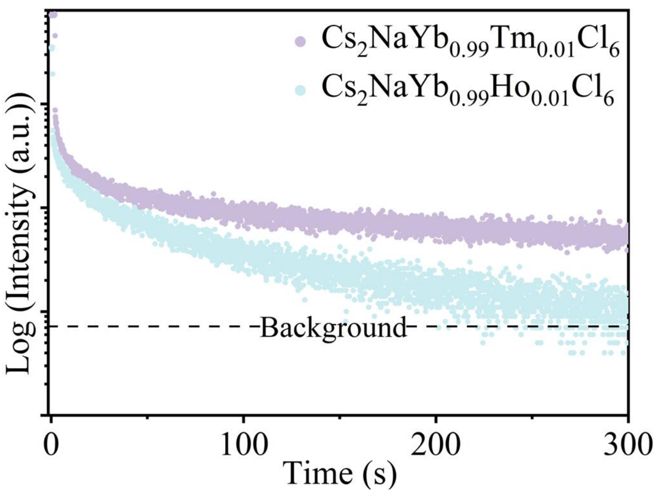

Figure S39. PersL decay curves of $Cs_2NaYb_{0.99}Tm_{0.01}Cl_6$ and $Cs_2NaYb_{0.99}Ho_{0.01}Cl_6$ NCs.

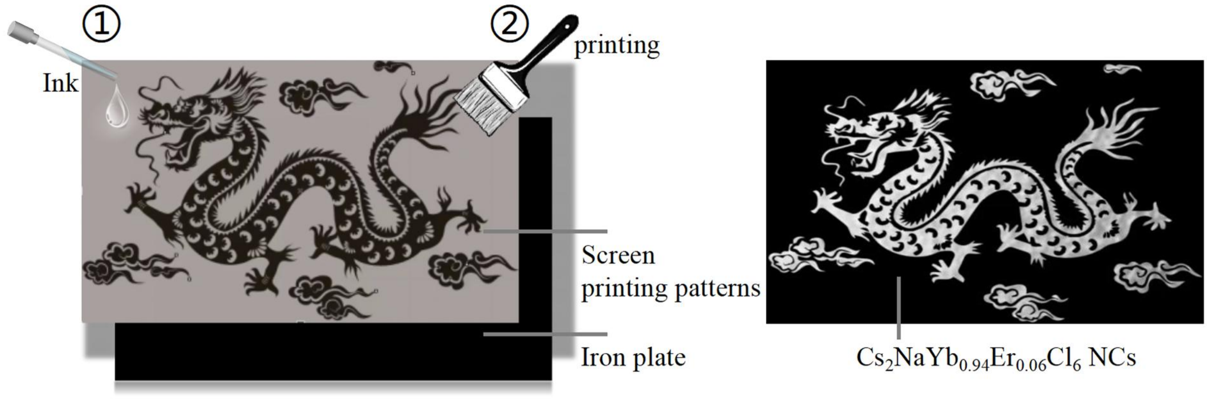

Figure S40. The screen printing process of preparing anti-counterfeiting patterns.

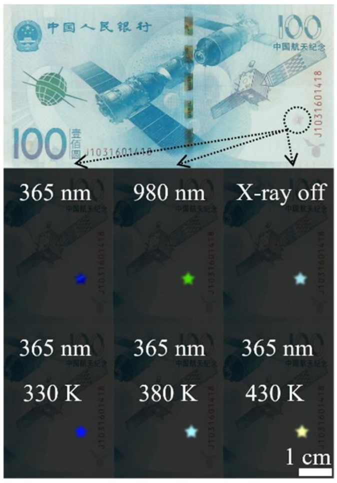

Figure S41. The "Star" is coated with $Cs_2NaYb_{0.94}Er_{0.06}Cl_6$ NCs on a 100 RMB commemorative coin, displaying different colors under various temperature and excitation lights.

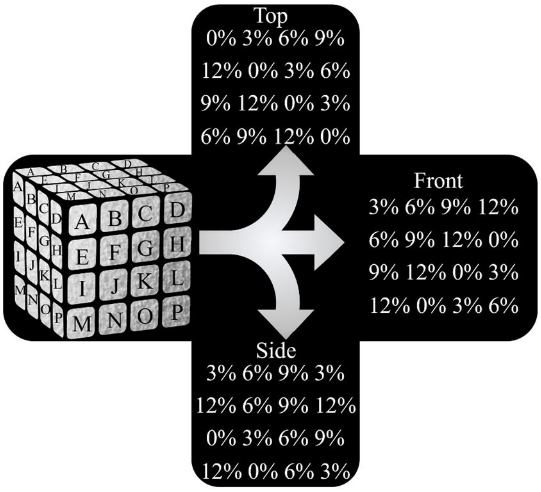

Figure S42. Photo of the cube code diagram show the front(A (3% Er), B(6% Er), C (9% Er), D (12% Er), E (6% Er), F (9% Er), G (12% Er), H (0% Er), I (9% Er), J (12% Er), K (0% Er), L (3% Er), M (12% Er), N (0% Er), O (3% Er) and P (6% Er)), side (A (3% Er), B(6% Er), C (9% Er), D (3% Er), E (12% Er), F (6% Er), G (9% Er), H (12% Er), I (0% Er), J (3% Er), K (6% Er), L (9% Er), M (12% Er), N (0% Er), O (6% Er) and P (3% Er)), and top (A (0% Er), B(3% Er), C (6% Er), D (9% Er), E (12% Er), F (0% Er), G (3% Er), H (6% Er), I (9% Er), J (12% Er), K (0% Er), L (3% Er), M (6% Er), N (9% Er), O (12% Er) and P (0% Er)).

References

[1] a) S. Burger, M. G. Ehrenreich, G. Kieslich, *J. Mater. Chem. A* **2018**, *6*, 21785; b)G. Kieslich, S. Sun, A. K. Cheetham, *Chem. Sci.* **2015**, *6*, 3430; c)V. M. Goldschmidt, *Naturwiss.* **1926**, *14*, 477.
